# Heat shock in *C*. *elegans* induces downstream of gene transcription and accumulation of double-stranded RNA

**DOI:** 10.1371/journal.pone.0206715

**Published:** 2019-04-08

**Authors:** Marko Melnick, Patrick Gonzales, Joseph Cabral, Mary A. Allen, Robin D. Dowell, Christopher D. Link

**Affiliations:** 1 Integrative Physiology, University of Colorado, Boulder, Colorado, United States of America; 2 BioFrontiers Institute, University of Colorado, Boulder, Colorado, United States of America; 3 BioFrontiers Institute and Department of Molecular, Cellular and Developmental Biology, University of Colorado, Boulder, Colorado, United States of America; 4 Institute for Behavioral Genetics, University of Colorado, Boulder, Colorado, United States of America; Universidad Nacional Autonoma de Mexico, MEXICO

## Abstract

We observed that heat shock of *Caenorhabditis elegans* leads to the formation of nuclear double-stranded RNA (dsRNA) foci, detectable with a dsRNA-specific monoclonal antibody. These foci significantly overlap with nuclear HSF-1 granules. To investigate the molecular mechanism(s) underlying dsRNA foci formation, we used RNA-seq to globally characterize total RNA and immunoprecipitated dsRNA from control and heat shocked worms. We find a subset of both sense and antisense transcripts enriched in the dsRNA pool by heat shock overlap with dsRNA transcripts enriched by deletion of *tdp-1*, which encodes the *C*. *elegans* ortholog of TDP-43. Interestingly, transcripts involved in translation are over-represented in the dsRNAs induced by either heat shock or deletion of *tdp-1*. Also enriched in the dsRNA transcripts are sequences downstream of annotated genes (DoGs), which we globally quantified with a new algorithm. To validate these observations, we used fluorescence *in situ* hybridization (FISH) to confirm both antisense and downstream of gene transcription for *eif-3*.*B*, one of the affected loci we identified.

## Introduction

Cytoplasmic proteotoxic stress induced by temperatures outside of the optimal range for cells or organisms triggers the heat shock response (HSR) [1]. The response to heat shock is multi-faceted and regulation of both transcription and translation occurs. Transcriptional responses include formation of stress granules, alternative splicing, and aberrant transcriptional termination [2–5]. The HSR is a highly conserved transcriptional response and is driven largely by the heat shock transcription factor HSF1 [6]. Under basal level conditions, HSF1 is a monomer in the cytoplasm and nucleus. Upon stress, HSF1 undergoes homotrimerization and binds to DNA heat shock elements (HSE) and initiates the transcription of heat shock protein genes [7,8]. In addition, translation of non-heat shock mRNAs is reduced through pausing of translation elongation as well as inhibition of translation initiation [9–11]. Regulation and clearance of misfolded proteins by heat shock proteins has been implicated in neurodegenerative diseases such as Huntington’s disease (HD), Parkinson’s disease (PD), Alzheimer’s disease (AD), and amyotrophic lateral sclerosis (ALS) [12].

Aside from the canonical binding of HSF1 to HSE loci, heat shock can cause HSE-independent transcriptional changes [2]. In mammalian cells, HSF1 granules colocalize with markers of active transcription where HSF1 binds at satellite II and III repeat regions [13]. In the worm *Caenorhabditis elegans*, HSF-1 (worm ortholog of HSF1) granules also show markers of active transcription but the putative sites of HSF-1 stress granule binding are unknown [14].

In addition to formation of HSF1 stress granules, heat shock can cause reduced efficiency of transcription termination and the accumulation of normally un-transcribed sequences, designated in the literature as downstream of gene containing transcripts (DoGs) [5]. In eukaryote transcriptional termination, the Carboxyl-terminal domain (CTD) of RNA Polymerase II (Pol II) interacts with a complex of cleavage and polyadenylation (CPA) factors responsible for generating the polyadenylate [Poly(A)] tail at mRNA 3’ ends. Two models exist for how the pre-mRNA poly(A) site (PAS) contributes to transcription termination. The allosteric model proposes that Pol II senses PAS during elongation leading to a conformational change in the Pol II active site eventually leading to Pol II release. The other, dubbed the torpedo model, proposes that the nuclear 5’-3’ exonuclease Xrn2 is recruited to the PAS and triggers Pol II release when it degrades the downstream transcript and catches up to elongating Pol II [15].

Recent studies have shown increased antisense transcription when read through transcription goes past the PAS into neighboring genes on opposite strands [16–19]. Antisense transcription has the potential to modulate gene expression by creation of double-stranded RNA (dsRNA) with subsequent degradation through RNA interference (RNAi) [20].

Previous studies in our lab found deletion of *tdp-1*, the worm ortholog of ALS associated protein TDP-43, results in the accumulation of dsRNA foci [21]. In addition to deletion of *tdp-1*, we discovered that heat shock robustly induced nuclear dsRNA foci in worms. To assay this unexpected formation of dsRNA, we performed strand-specific RNA-seq and strand-specific RNA immunoprecipitation sequencing (RIP-seq) with the J2 antibody specific for dsRNA. In heat shocked worms, we find increased J2 enrichment of downstream of gene transcripts as well as genes involved in translation. To identify altered transcription genome-wide, we developed an algorithm called Dogcatcher that identifies DoG locations, genes that overlap with DoGs on the same or opposite strand, and an optional pipeline to provide differential expression of DoGs.

## Materials and methods

### *Caenorhabditis elegans* culturing and strains

Hermaphrodites from each strain were kept at 16 °C on Nematode Growth Media (NGM) plates seeded with Escherichia coli strain OP50 as a food source according to standard practices [22]. To obtain age synchronized worms, we used alkaline hypochlorite bleach on gravid adults to obtain eggs that were hatched overnight in S-basal buffer [23]. Worms were then allowed to grow to 1 day old adults (approximately 80h at 16 °C). List of strains used in this study is available in S1 Table.

### Heat stress treatment

Heat stress treatment was performed in an air incubator set to 35 °C for 3 hours for the RNA-seq experiments. After stress, populations were washed off with S-basal buffer and immediately fixed for immunohistochemistry or fluorescence in situ hybridization (FISH), flash frozen in liquid nitrogen for quantitative reverse transcriptase polymerase chain reaction (qRT-PCR), or crude extracts were created with subsequent J2 Immunoprecipitation (J2 IP) as previously described [21].

### RNA isolation, cDNA library preparation, and RNA sequencing

Total RNA was extracted from worms using TRIzol (Invitrogen #15596026) extraction and used as input RNA. Chloroform was used to solubilize proteins and TURBO DNAase (Invitrogen) was used to remove DNA. For input RNA libraries, 5 μg of RNA was ran through a RiboZero column (Epicenter, #R2C1046) to remove ribosomal RNA. Libraries were created using Illumina TruSeq kits (RS-122-2001). RNA recovered by immunoprecipitation with the J2 antibody of young adult worms as well as input material (as a loading control) was converted into strand-specific total RNA libraries using V2 Scriptseq (Epicenter #SSV21106) kits following manufacturer's instructions, except reverse transcription was done with SuperScript III (Invitrogen #18080 044) using incrementally increasing temperatures from 42 to 59 °C to allow for transcription though structured RNAs. rRNA was not removed from J2 IP RNA samples. Libraries were sequenced on an Illumina HiSeq 2000 platform at the Genomics Core at the University of Colorado, Denver. Data were deposited under GEO accession number GSE120949.

### Immunohistochemistry and fluorescence in situ hybridization (FISH)

For immunohistochemistry, all washes used a constant volume of 1ml sterile S-basal buffer unless otherwise noted. Worms were first washed off plates, spun down into a pellet, and fixed in 4% paraformaldehyde. Worms were then resuspended in 1ml of Tris-Triton buffer with 5% beta-mercaptoethanol and incubated in a rocker for two days at 37°C. After two days, worms were washed two times and put into collagenase buffer. Next, worms were placed into a 1:1 dilution of 1mg/ml type IV collagenase (Sigma) and S-basal buffer for 45 minutes at 37 °C with rocking. Worms were checked under the microscope to ensure cuticle breakage then quenched in cold Antibody buffer A (1X Phosphate buffered saline, 0.1% Bovine Serum Albumin, 0.5% Triton X-100, 0.05% Sodium Azide). Worms were then washed, pelleted, and primary antibodies were added for 16 hours at 4°C. Next, worms were washed twice in Antibody buffer B (same as Antibody buffer A except using 1% Bovine Serum Albumin), pelleted, and secondary antibodies were added with subsequent incubation for 2 hours at room temperature. Finally, worms were washed twice in Antibody buffer B and then placed in 50ul of Antibody buffer A. Permeabilized worms were probed with the primary J2 antibody (English and Scientific Consulting Lot: J2-1102 and J2-1103) at 4μg/mL and secondary antibody Alexa dye-conjugated goat anti-mouse at 4μg/mL. DAPI nuclear stain was added along with secondary antibodies at 5μg/mL to visualize nuclei.

Stellaris FISH probes (Biosearch technologies) [24] were custom designed using the Stellaris RNA FISH probe designer. Three regions were chosen for probing, and each probe was tested against the *C*. *elegans* genome using BLAST to identify any complementarity to non-target sequences. A probe was excluded if it was in an intron, had a highly repetitive sequence outside of the region, or matched other regions up to 18nt long with high transcriptomic expression viewed in the Integrative Genome Viewer (IGV) [25]. Probes and locations are available in S1 File.

For FISH probing and storage, the Stellaris protocol for *C*. *elegans* was followed using RNAase OUT (Invitrogen) when applicable. Briefly, worms were washed off plates using nuclease-free water and fixed for 45 minutes at room temperature in a fixation buffer (1:1:8 of 37% formaldehyde, 10X RNAase-free phosphate buffered saline (PBS), nuclease-free water). Worms were then washed twice with 1X RNAase-free PBS and permeabilized in 70% ethanol overnight at 4°C. Worms were then incubated at room temperature in Stellaris Wash Buffer A, pelleted, and incubated for 16 hours in a 37 °C water bath in the dark with 100μl of the hybridization buffer (9:1 of μl Stellaris RNA FISH hybridization buffer, deionized formamide with a 100:1 Hybridization buffer, FISH probe). Next, 1mL of Stellaris Wash Buffer A was added with 30 more minutes of incubation in the dark 37 °C water bath. Stellaris Wash Buffer A was then aspirated and incubated with DAPI (1:1000 of 5μg/ml DAPI, Stellaris Wash Buffer A) for 30 more minutes of the dark 37 °C water bath. Lastly, the DAPI buffer was aspirated and 1mL of Stellaris Wash Buffer B was added with a 5 minute room temperature incubation.

A modification of the immunohistochemistry protocol was used when doing immunohistochemistry and FISH. The immunohistochemistry protocol was the same except all washes were done using RNAase-free PBS or water and RNAase-free reagents (Tris-Triton buffer, collagenase buffer, collagenase, Antibody Buffer A, Antibody Buffer B) were created by adding RNAase OUT (2:10000 of RNAase OUT, reagent). After antibody staining, the FISH protocol was started at the hybridization step.

### Microscopy

Images were acquired with a Zeiss Axiophot microscope equipped with digital deconvolution optics (Intelligent Imaging Innovations). Image brightness and contrast were digitally adjusted in Photoshop.

### Quantification of coincidence of J2 and HSF-1 foci over time and J2 foci in mutant strains

**Fig 1:**
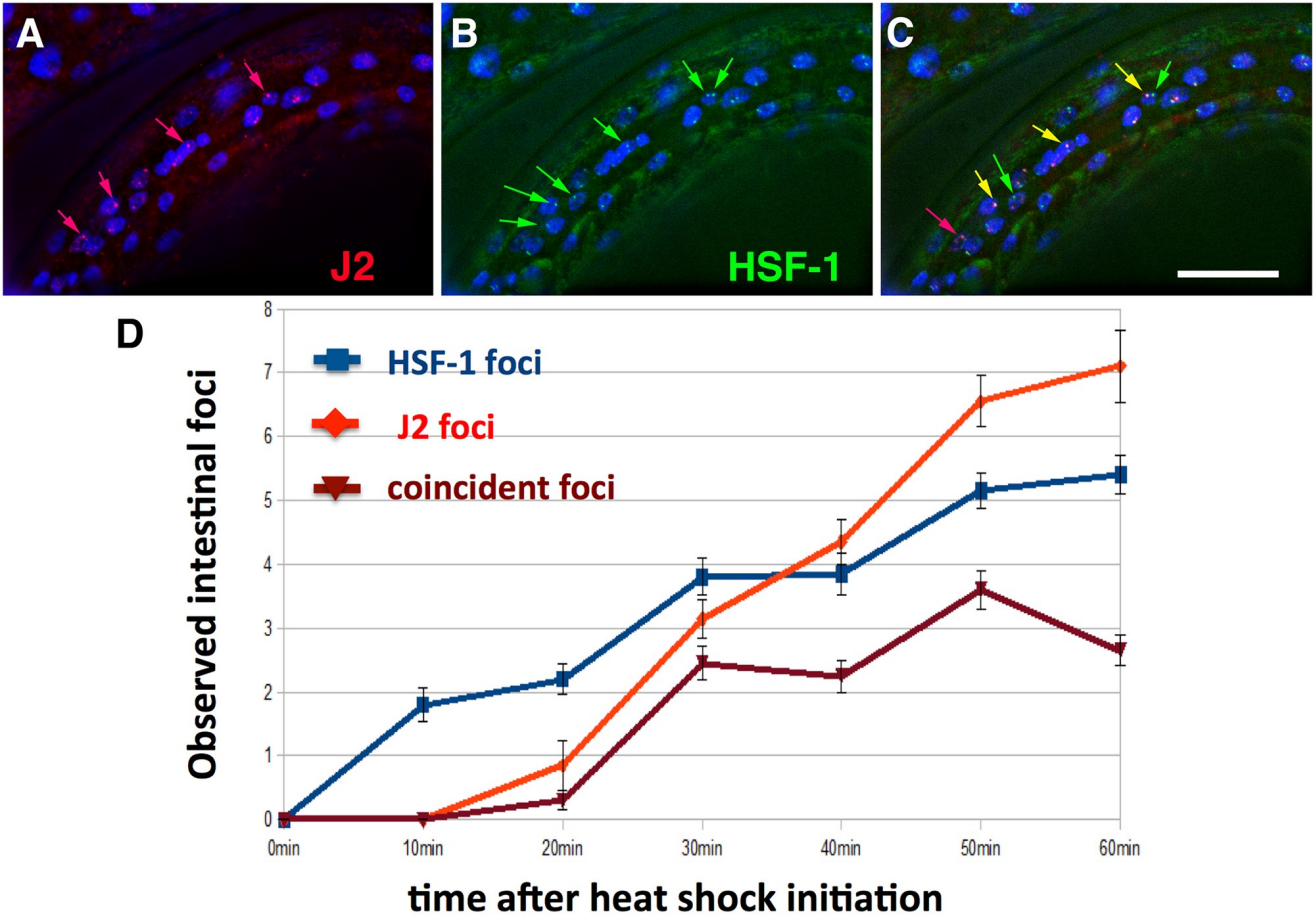
Heat shock induces nuclear foci detectable with dsRNA-specific antibody J2. Mid-animal intestinal region of 4th larval stage drSI13 worm fixed 40 minutes after heat shock at 35° C. (A) Nuclear J2 foci (red arrows). (B) HSF-1 foci (green arrows). (C) Overlap of J2 foci and HSF-1 foci (orange arrows). White size bar in bottom right corner (20 microns across). DNA stained with DAPI (blue). (D) Quantification of occurrence of HSF-1 and J2 foci over time. 19–20 worms scored per time point with 4 intestinal nuclei scored per worm.

For the quantification of coincidence of foci over time, intestinal nuclei of the worms were isolated from the rest of the image and the Foci Picker3D plug-in was used to count foci. The FITC channel of the image was converted to 16 bit and analyzed. Foci Picker3D settings were changed from default by changing the Minlsetting to 0.25 and the ToleranceSetting to 20 before running analysis. 19–20 worms were selected for each time point. Analysis of variance (ANOVA) and Tukey honestly significant difference (HSD) post-hoc analysis were used for multiple comparisons between conditions with a significance threshold of < 0.05 (Family Wise Error Rate). The script for the ANOVA and post-hoc is available at https://github.com/Senorelegans/heatshock_and_tdp-1_dsRNA_scripts/tree/master/fig1_coincidents_of_foci/fig_supplemental_graph_foci.

Quantification of J2 foci in mutant strains with or without heat shock was done blindly. 8 worms per condition were counted and the average number of foci from 2–4 intestinal nuclei were used to make the box plots. Analysis of variance (ANOVA) and Tukey HSD post-hoc analysis were used for multiple comparisons between conditions with a significance threshold of < 0.05 (Family Wise Error Rate). The script for creating boxplots and ANOVA and post-hoc is available at https://github.com/Senorelegans/heatshock_and_tdp-1_dsRNA_scripts/tree/master/fig_supplemental_graph_foci.

### Sequencing data analysis

Detailed instructions on algorithms and analysis is provided in S3 File. Briefly, reads were checked for quality with FastQC v0.11.7 [26], adapters were trimmed using Trimmomatic-0.36 [27] (S2 File), and reads were aligned to the worm genome WS258 using STAR-2.5.2b [28]. Genes and DoGs (identified by Dogcatcher, described below) were assigned counts using Rsubread v1.28.1 featureCounts [29] and were rRNA normalized according to the rRNA subtraction ratio (RSR) (described in supplemental). Differential expression was obtained using DESeq2 v1.20.0 and the likelihood ratio test (LRT) set with input and J2 groups treated as separate variables within the condition [30].

We created an algorithm called Dogcatcher to identify and analyze DoGs. Briefly, Dogcatcher uses a sliding window approach to identify contiguous regions of transcription above a defined threshold. If the sliding window runs into a gene on the same strand it will either continue (meta read through) or stop (local read through). Dogcatcher outputs bedfiles, gtf’s and dataframes of all DoGs and antisense DoGs identified within a sample along with differential expression and genes overlapping DoGs (For additional details see bioinformatics supplemental S3 File). For improved normalization in DESeq2, non-significant genes are added when calculating differential expression and removed for visualization. The Dogcatcher algorithm and README is available at https://github.com/Senorelegans/Dogcatcher. For processing J2 enrichment, a modified version of Dogcatcher was used that applies the likelihood ratio test from DESeq2 (available at https://github.com/Senorelegans/heatshock_and_tdp-1_dsRNA_scripts/J2_enrichment_Dogcatcher).

DoGs identified by Dogcatcher that overlap operons on the same strand were removed (S3 File for operon removal methods).

All of the scripts used to process the data and create figures can be found at https://github.com/Dogcatcher/heatshock_and_tdp-1_dsRNA_scripts.

## Results

### Heat shock induces nuclear dsRNA foci in *C*. *elegans*

While looking for conditions that might induce dsRNA foci besides loss of *tdp-1*, we found that heat shock robustly induced dsRNA nuclear foci. Upshifting wild type worms to 35 °C or 37 °C induced foci detectable with the J2 dsRNA-specific monoclonal antibody within 30 minutes, primarily visible in intestinal and hypodermal nuclei. To determine if these foci overlapped with previously identified nuclear HSF-1 stress granules, we repeated the heat shock experiment with strain OG497 (*drSI13*) [14]. This strain has a single copy insertion of *hsf-1* with a C-terminal GFP driven by the *hsf-1* promoter, and shows nuclear GFP expression that redistributes into granules after a one minute heat shock at 35 °C [14]. Using the J2 antibody for immunohistochemistry, we found J2 dsRNA foci in nuclear regions that partially overlapped with nuclear HSF-1 stress granules when drSI-13 worms were heat shocked for 35 °C for 40 minutes (Fig 1A–1C). Measuring coincidence of foci over one hour in 10 minute increments, we observe a significant change [Family Wise Error Rate (FWER) < 0.05] for all time points 30–60 minutes compared to 10 minutes. (Fig 1D) (S2 Table for raw data, Material and methods).

Since dsRNA foci partially overlap with HSF-1 stress granules, we were curious if a HSF-1 partial loss of function mutant *hsf-1(sy441)* [31] would change the amount of dsRNA foci present. We found no significant (FWER < 0.05) differences upon heat shock in the amount of intestinal J2 foci in the *hsf-1(sy44)* mutant compared to heat shocked wild type (S1 Fig, Material and methods for statistical analysis of S1 Fig).

Similar to the *hsf-1(sy441)* mutant, we looked for any effect upon dsRNA formation in a *rde-4(n337)* knockout strain. RDE-4 is a double-stranded RNA binding protein (dsRBP) required for the initiation of RNA interference (RNAi) in *C*. *elegans* [32]. We found no significant differences (FWER < 0.05) between heat shocked *rde-4(n337)* and heat shocked wild type strains, although we found low levels of dsRNA foci in non-heat shocked *rde-4* (S1 Fig).

### Recovery of dsRNA by J2 immunoprecipitation

In order to identify dsRNA transcripts induced by heat shock, we performed strand-specific RNA sequencing (RNA-seq) and strand-specific RNA immunoprecipitation sequencing (RIP-seq) (S2 Fig). Input RNA and RNA immunoprecipitated with the J2 antibody was extracted and sequenced for heat shocked N2 (wild type) worms (in duplicate) and non-heat shocked worms (in triplicate). The J2 antibody is specific for dsRNA 40bp or more [33] and transcripts from the J2 Immunoprecipitation (IP) could include full length dsRNA transcripts or single stranded RNA (ssRNA) adjacent to 40bp or more sections of dsRNA. dsRNA can occur via base pairing with a different transcript (interstrand) or self-complementarity within the same transcript (intrastrand). Similar to previous experiments, RNA immunoprecipitated samples were normalized to input RNA samples [21,34].

### Measurement of antisense gene transcripts after heat shock

**Fig 2:**
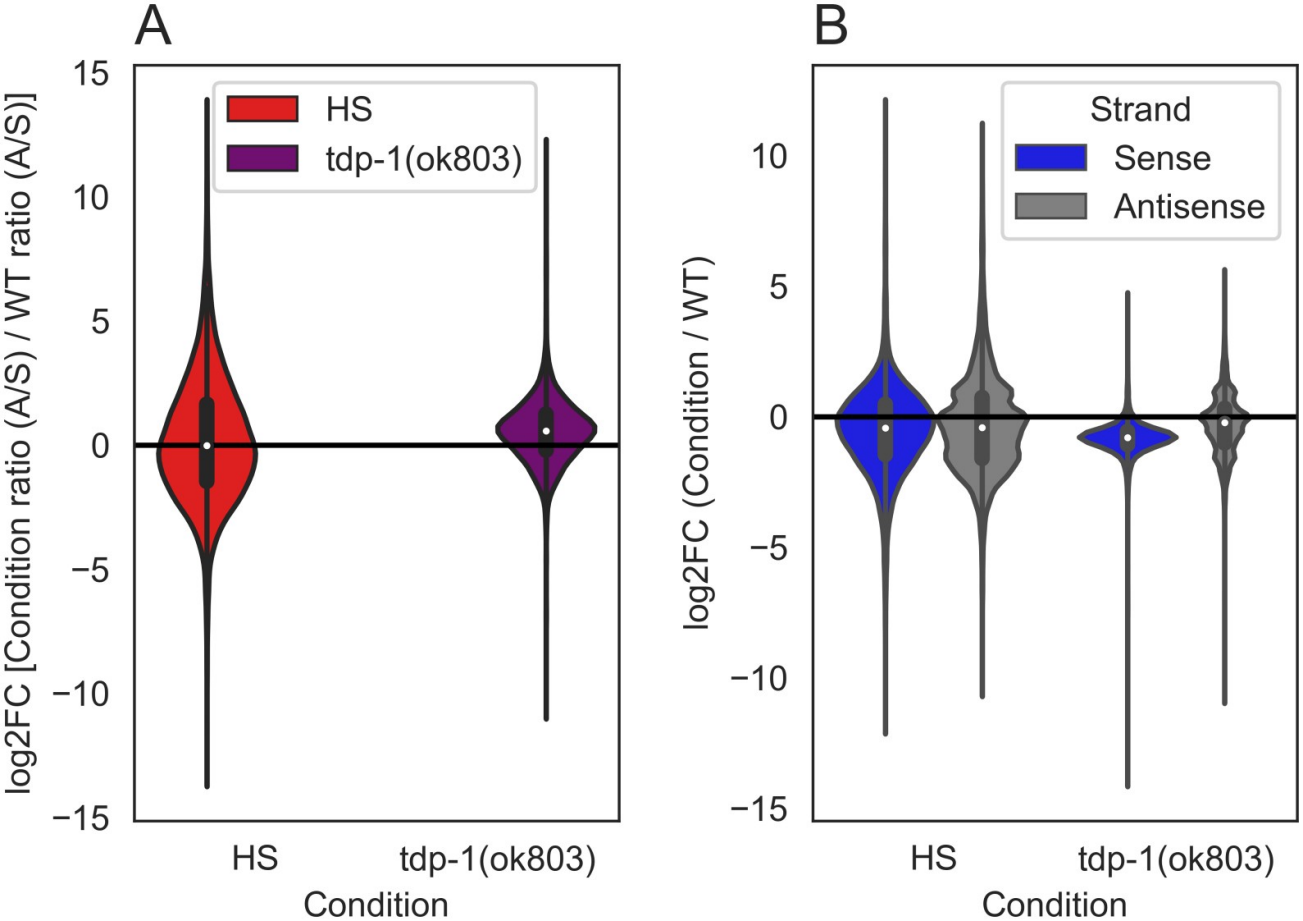
Quantification of genes with changes in antisense/sense ratios after heat shock or deletion of *tdp-1* in input RNA. Violin plots of the ratio of read counts over gene regions for each condition compared to wild type (WT) [mean >20 for pooled counts (sense and antisense, condition and wild type), n = 1]. (A) With heat shock 5513/11091 genes have a higher antisense/sense ratio compared to wild type (log2FC > 0, area of red violin plot above the black line). With *tdp-1(ok803*) 7551/10831 genes have a higher antisense/sense ratio compared to wild type (log2FC > 0, area of purple violin plot above the black line). (B) With heat shock, 4125/11091 sense and 3679/11091 antisense transcripts are upregulated compared to wild type (log2FC > 0, area of violin plot above the black line). With *tdp-1(ok803*) 419/10831 sense and 3301/10831 antisense transcripts are upregulated compared to wild type.

The apparent increase in dsRNA we observed in heat shocked worms [and previously observed in the *tdp-1*(*ok803*) mutant] could result from an increased accumulation of antisense transcripts. To obtain a global view of antisense levels, we calculated an antisense/sense ratio for genes using the input RNA samples (S3 File for methods). For a stringent view of fold changes between conditions, genes with a minimum of 20 mean read count (sense and antisense pool) between condition and wild type were used for this analysis. Out of 46760 worm genes, using this cutoff we scored 11091 genes in heat shock compared to wild type, and 10831 genes in *tdp-1(ok803)* compared to wild type. When we look at antisense/sense ratios of read counts over genes for each condition compared to wild type (Fig 2A), we find no difference in the ratio with heat shock (5513/11091 ~49.70%), and an increase in the ratio (7551/10831 ~69.71%) with the *tdp*-1 deletion. Since antisense/sense ratios can increase either through depletion of sense transcripts or increases in antisense transcripts, we examined sense and antisense levels separately in each condition compared to wild type (Fig 2B). With heat shock, we find no increase [log2 fold change (log2FC) > 0] in sense (4125/11091 ~37.91%) or antisense (3679/11091 ~33.79%) transcripts. With the *tdp*-1 deletion, however, we find noticeably fewer genes with increased sense counts (419/10831 ~3.86%) and no increase in antisense counts (3301/10831 ~30.47%) over genes compared to wild type. Thus the increase in antisense/sense ratio in the *tdp-1* deletion arises because of lowered accumulation of sense transcripts rather than increased antisense transcript levels. This was not unexpected as TDP-1 plays a role in normal transcription [21].

To further validate this result, we analyzed the total RNA-seq data of Brunquell et al [35], who performed similar heat shock experiments in *C*. *elegans*. In the Brunquell dataset we found a small increase in the amount of genes with significant antisense transcription upon heat shock (28/1818 ~1.54%) compared to no heat shock in wild type worms (S2 Fig and S3 File for bioinformatic methods). We conclude that in *C*. *elegans* heat shock does not result in transcriptional dysregulation that leads to a large increase in antisense transcripts.

### Comparison of dsRNAs identified in worms heat shocked or deleted for *tdp-1*

**Fig 3:**
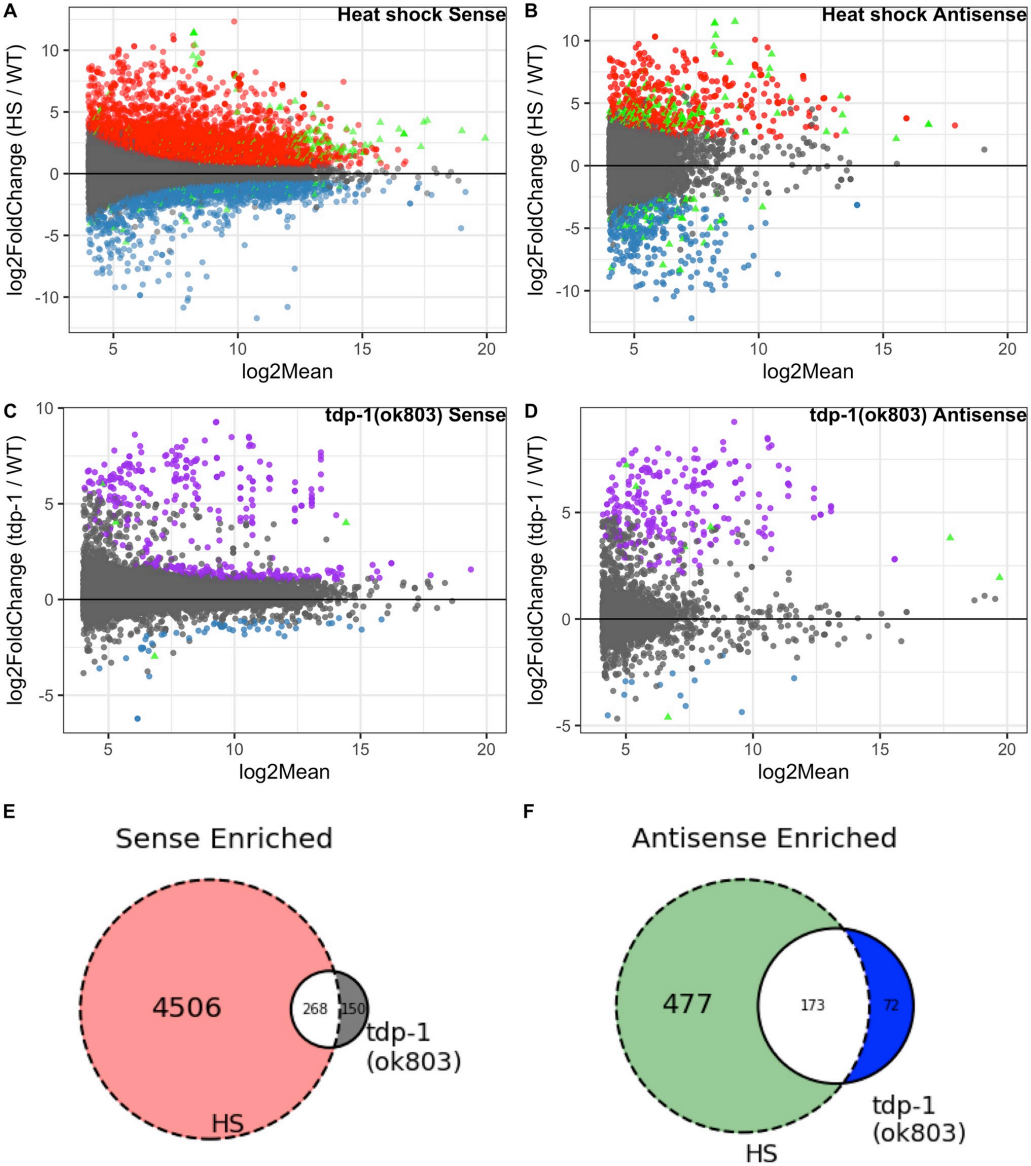
Comparison of J2 enriched sense and antisense transcripts in heat shock and *tdp-1(ok803)* worms. MA plots [“M” (log2FC) on y-axis and “A” (log2Mean) on x-axis] of significant (FDR <0.05) dsRNA enrichment for sense and antisense transcripts (analyzed independently) along with Venn diagrams of enrichment for enriched sense and antisense [n = 2 for heat shock J2 samples, n = 3 for wild type, n = 3 for *tdp-1(ok803)*]. (A) Heat shock over wild type J2 enriched sense transcripts with 4774 enriched (red) and 1669 depleted (blue). (B) Heat shock over wild type J2 enriched antisense transcripts with 650 enriched (red) and 477 depleted (blue). (A-B) enriched (180) and depleted (48) heat shock vs wild type transcripts found in both sense and antisense (green triangles). C: *tdp-1(ok803)* over wild type significant J2 enriched sense transcripts with 418 enriched (purple) and 59 depleted (blue). (D) *tdp-1(ok803)* over wild type significant J2 enriched antisense transcripts with 245 enriched (purple) and 14 depleted (blue). (C-D) enriched (6) and depleted (1) *tdp-1(ok803)* vs wild type transcripts found in both sense and antisense (green triangles). (E) Overlap of genes with significantly J2 enriched sense transcripts in both conditions compared to wild type worms. (F) Overlap of genes with significantly J2 enriched antisense transcripts in both conditions compared to wild type worms.

Considering that both heat shock and deletion of the *tdp-1* gene lead to the formation of nuclear dsRNA foci, we sought to determine if this phenotypic similarity also extends to transcripts that are accumulating in the dsRNA pool. After heat shock, we found a large number of significant gene transcripts [false discovery rate (FDR) < 0.05 and a log2 mean expression (log2Mean) > 4]. Specifically, in the pool of RNAs immunoprecipitated by the dsRNA-specific antibody J2 (relative to untreated worms), we found (4774/18737) significantly enriched or (1669/18737) significantly depleted transcripts (Fig 3A). We also identified antisense transcripts with significantly altered representation in the J2 IP pool, and found 650/8832 enriched and 477/8832 depleted (Fig 3B). A minority of genes had both sense and antisense transcripts significantly enriched (180) or depleted (48) in the heat shock J2 IP pool (Fig 3A and 3B) (plot of genes showing only significant sense and antisense transcription for heat shocked worms in S4 Fig). In *tdp-1(ok803)* significant (FDR <0.05, log2Mean > 4) gene transcripts, we found a smaller number of sense enriched (418/13223) and depleted (59/13223) (Fig 3C), as well as antisense enriched (245/2343) and depleted (14/2343) genes (Fig 3D). Similar to heat shock, *tdp-1(ok803)* had relatively fewer genes with both sense and antisense transcripts significantly enriched (6) and depleted (1) (Fig 3C and 3D) (plot of genes showing only significant sense and antisense transcription for *tdp-1(ok803)* worms in S5 Fig). We found a significant [P < 1 x 10^−30^, hypergeometric distribution (hgd)] overlap of J2 enriched gene transcripts between the heat shock and *tdp-1*(*ok803*) populations in both sense (Fig 3E) and antisense (Fig 3F), suggesting that there might be some similarities between the dsRNA accumulation induced by heat shock and deletion of *tdp-1*. However, with J2 depleted transcripts, we found no significant overlap in (P = 0.165, hgd) in sense transcripts and no significant overlap in antisense transcripts (P = 0.187, hgd) (See supplemental S4 File for list of genes and hgd implementation).

We next sought to examine whether the dsRNAs arising in heat shock or *tdp-1(ok803)* showed enrichment for similar pathways. Using GOATOOLS [36], we found that many Gene ontology (GO) terms related to translation were significantly enriched (FDR < 0.05) in both the heat shock and *tdp-1*(*ok803*) J2 IP pools. Out of 330 translation related genes classified by GOATOOLS, in sense J2 enriched transcripts, we find 234 translation related genes with heat shock, 27 translation related genes in *tdp-1(ok803)*, and 19 translation related genes in the overlap (S3 File for methods). In the J2 depleted sense pool, only heat shocked worms contained translation related genes (30 total) with none in *tdp-1(ok803)* pool. In J2 enriched antisense transcripts, only heat shocked worms had 33 translation related genes. There was no translation related genes found in J2 depleted antisense transcripts (S5 File for list of significant genes and translation related genes, S6 File for GOATOOLS output). Thus the dsRNA recovered by J2 immunoprecipitation is enriched for translation related pathways under both conditions, but there are distinct transcripts in heat shock compared to *tdp-1(ok803)* worms.

### Enrichment of transcripts downstream of genes in the J2 pool

**Fig 4:**
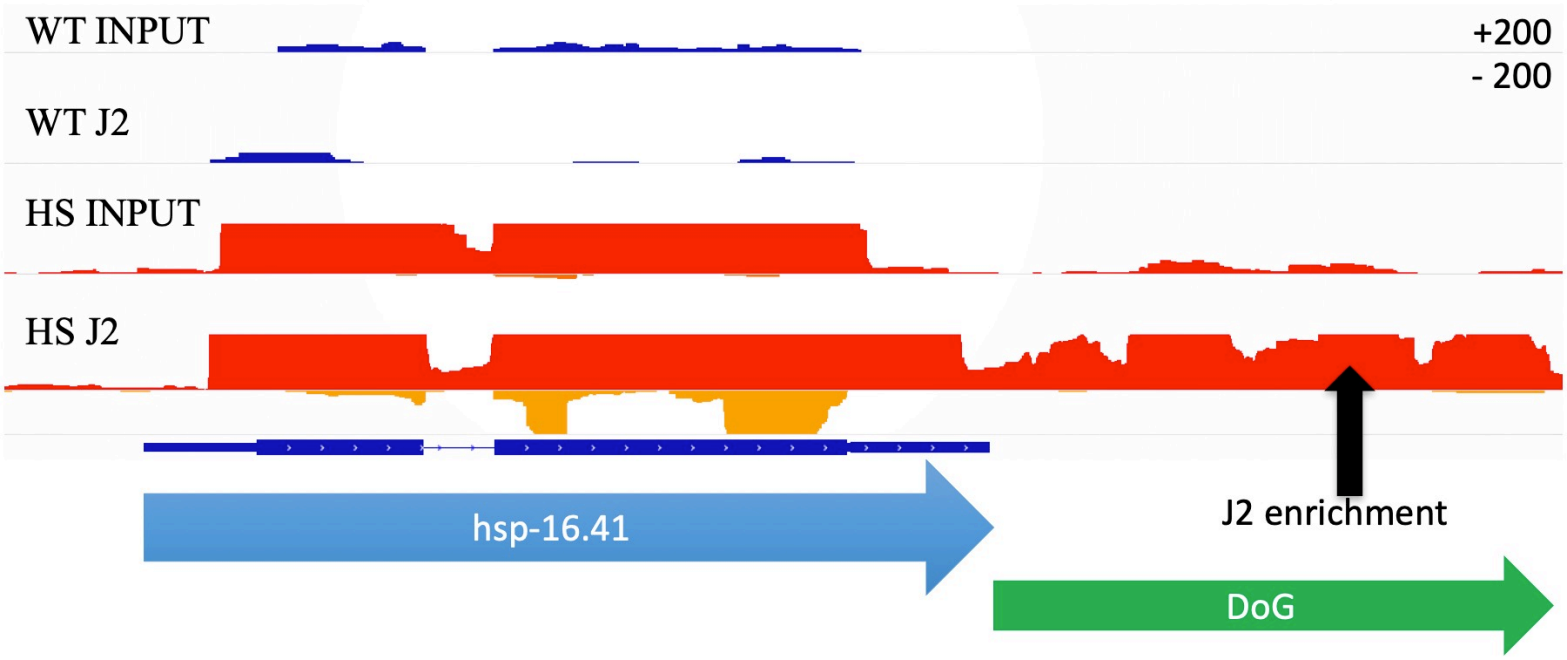
Aberrant transcription past the end of heat shock family genes showed enrichment in heat shock J2. Normalized histogram from the Integrative Genomics Viewer (IGV). On each track, the sense strand is on the top part of the histogram and antisense is on the bottom (Max read depth +/- 200). Wild type (WT) sense (dark blue) and antisense (light blue), heat shock (HS) sense (red) and antisense (orange). Gene transcription continues past the 3’ end of gene (blue arrow) in heat shock, leading to an annotated downstream of gene transcript (DoG) (green arrow).

While examining the transcription of known heat shock inducible genes, we noted in heat shocked populations an accumulation of read through transcripts downstream of annotated genes (see example in Fig 4). Interestingly, some of these downstream of gene transcripts (DoGs) were also highly enriched in the J2 IP pool. While previous work has characterized the accumulation of downstream of gene transcripts in heat shocked cells from human [5] and mice [16], the phenomena has not previously been associated with dsRNA accumulation. To annotate read through regions across the whole genome, we created an algorithm called Dogcatcher. Dogcatcher uses a sliding window approach (100 bps) to annotate read through regions. In addition to Dogcatcher, we established an optional wrapper for quantifying differential expression through Rsubread and DESeq2 (See methods and materials as well as GitHub README for algorithm explanation).

Using the Dogcatcher algorithm in conjunction with the *C*. *elegans* genome annotation to identify DoGs *de novo*, we were able to quantify downstream of gene transcripts in the J2 IP pool that would be missed using the standard *C*. *elegans* genome annotation. Differential expression of transcription in downstream of gene regions can occur via novel DoGs that are in one sample and not another, or by varying levels of transcription of a DoG that is expressed in both samples. Our analysis suggests that both mechanisms may be involved. When we compare heat shocked worms to wild type in the J2 IP and input RNA, we find the majority of annotated DoGs (272/490 ~56%) come from the J2 IP from heat shocked worms (S6A Fig). When we compare *tdp-1(ok803)* worms to wild type in the J2 IP and input RNA, we find that the largest fraction comes from input RNA from *tdp-1(ok803)* worms (85/300 ~28%) followed closely by DoGs that are shared between J2 IP and input RNA for both wild type and *tdp-1(ok803)* worms (52/300 ~17%) (S6B Fig). This suggests that with heat shock the majority of DoGs are novel dsRNA enriched regions, in contrast to the *tdp-1* deletion which has a bigger overlap with DoGs in wild type samples.

**Fig 5:**
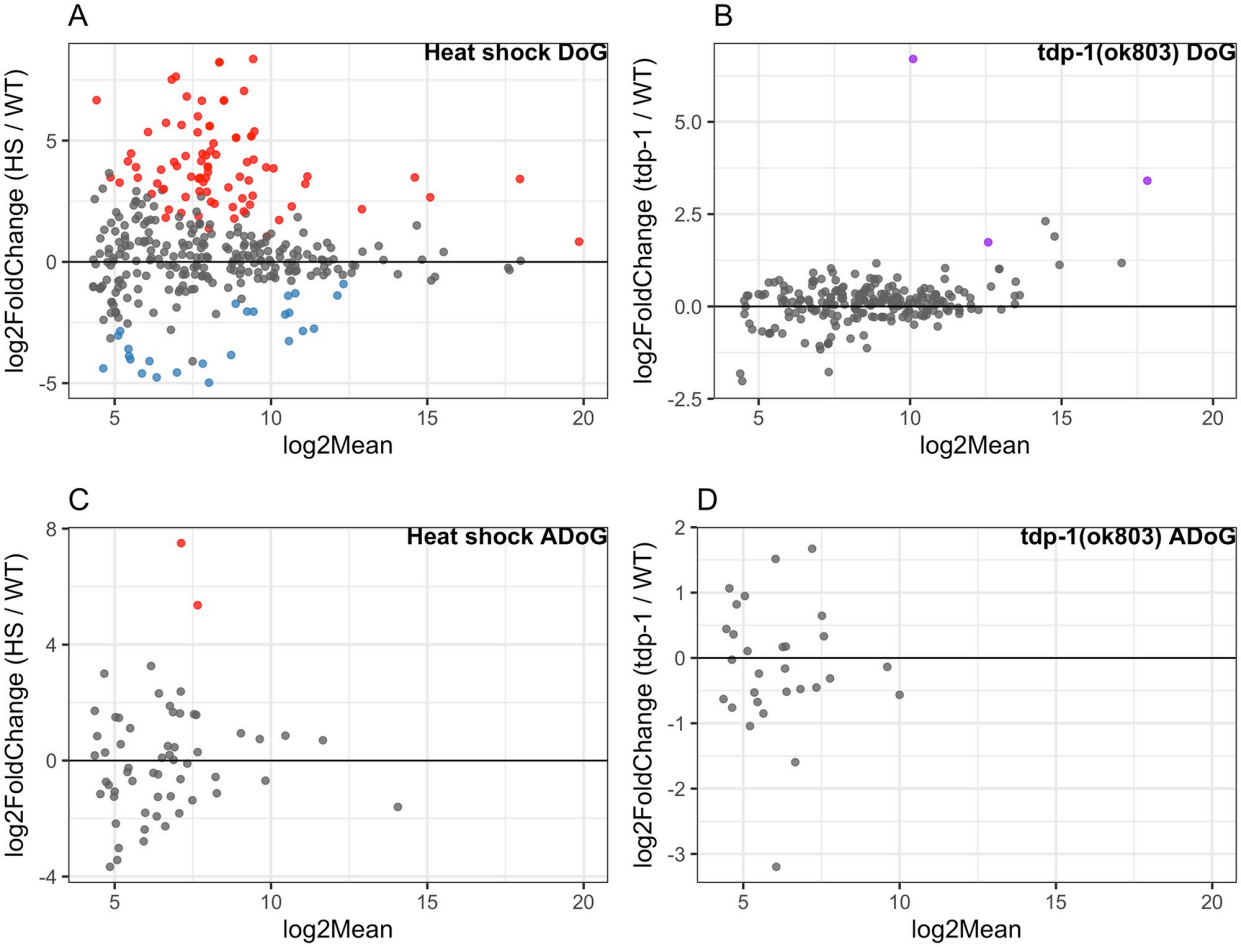
J2 enrichment of DoGs and ADoGs in heat shock and *tdp-1(ok803)* worms. MA plots of significant (FDR <0.05) dsRNA enrichment for DoGs and ADoGs. Annotated genes that were not significantly changing were added in with DoGs or ADoGs for DESeq2 normalization but were taken out of the plots for clarity [n = 2 for heat shock J2 samples, n = 3 for wild type, n = 3 for *tdp-1(ok803)*]. (A) Heat shock over wild type J2 enriched read through sense transcripts with 84 enriched (red) and 25 depleted (blue) out of 421 scored DoGs. (B) *tdp-1(ok803)* over wild type significant J2 enriched read through sense transcripts with 3 enriched (purple) out of 265 scored DoGs. (C) Heat shock over wild type J2 enriched read through antisense transcripts with 2 enriched (red) out of 70 scored ADoGs. (D) No significant *tdp-1(ok803)* over wild type J2 enriched read through antisense transcripts out of 43 scored ADoGs.

To quantify differential expression, we used the Dogcatcher optional differential expression wrapper. After heat shock, more read through sections were significantly (FDR <0.05, log2Mean > 4) enriched in the J2 IP pool than depleted (84 vs. 25 out of 421) (Fig 5A). Of the 84 DoGs significantly increased in the J2 IP pool with heat shock, the largest group of DoGs (35/84 ~41.66%) are only present in the J2 IP (S6C Fig). Of the 25 DoGs significantly decreased in the J2 IP with heat shock, we find a fairly even split between groups (S6D Fig). We found that for DoGs enriched in the J2 IP pool after heat shock, the majority corresponds to protein coding genes (60%), followed by non-coding RNA (ncRNA) (20%), pseudogenes (9%), and small nucleolar RNA (snoRNA) (9%). When we looked at significant sense genes with corresponding significant DoGs upon heat shock, we found 62 out of 84 enriched and 11 out of 25 depleted DoGs in the J2 IP have corresponding significant genes (S7A and S7B Fig). Consistent with our results, we found a small increase in the amount of significant (FDR <0.05, log2Mean > 4) DoGs upon heat shock (23/488 ~4.7%) compared to wild type (2/488 ~0.4%) in the Brunquell et al [35] dataset (S8 Fig, S7 File for list of DoGs, and S3 File for methods).

We found far fewer significantly (FDR <0.05, log2Mean > 4) J2 enriched DoGs from *tdp-1(ok803)* (3 out of 265) with no regions being depleted (Fig 5B). Interestingly, 2 out of the 3 DoGs in *tdp-1(ok803)* were also enriched in the heat shock J2 pool (S4 File for list DoGs and hgd implementation). When we looked at significant sense genes with corresponding significant DoGs upon *tdp-1* deletion, we found 1 out of 3 DoGs enriched in the J2 IP have corresponding significant genes (S7C Fig).

From the significantly enriched GO terms of DoGs in heat shock and *tdp-1(ok803)* worms, only heat shocked worms had any significantly enriched GO terms, which primarily consisted of histone genes (S7 File for list of DoGs, S8 File for GOATOOLS output). As a possible explanation for the formation of dsRNA at downstream of gene regions, we found DoGs to be enriched in terminal repeat sequences compared to a random intergenic downstream background. (S8 Fig, S3 File for methods).

### Additional non-annotated transcripts are minimally enriched in the J2 pool after heat shock or *tdp-1* deletion

Next, we were curious if other sections around genes would show aberrant transcription in heat shock or *tdp-1(ok803)* worms. Expanding on the DoG nomenclature, the terms we use for the three other types of transcription flanking an annotated gene are as follows: regions downstream of genes with antisense reads (ADoGs), sense reads in regions previous of the gene (PoGs), and antisense reads in regions previous of the gene (APoGs) (See S10 Fig for visualizations and additional explanation). Importantly, novel areas of intergenic transcription are obtained by filtering out PoGs with any overlap to DoGs on the same strand, as well as ADoGs or APoGs with any overlap to DoGs (or genes) on the opposite strand (See S10 and S11 Figs for visualization of filtering). We did not find any significantly (FDR <0.05, log2Mean > 4) J2 enriched PoGs or APoGs in either condition compared to wild type. We found a small amount of significant (FDR <0.05, log2Mean > 4) J2 enrichment in heat shock ADoGs (2 out of 70) (Fig 5C) and no ADoGs enriched in *tdp-1(ok803)* out of 43 scored ADoGs (Fig 5D).

Consistent with our heat shock results, in our analysis of Brunquell et al [35], we found a small increase in the amount of significant (FDR <0.05, log2Mean > 4) ADoGs upon heat shock in wild type worms (3/51 ~5.88%) and no significant ADoGs without heat shock. (S9 Fig, S7 File for list of ADoGs and S3 File for bioinformatic methods).

### Increased antisense transcription over genes associated with DoGS and ADoGS

Next, we were curious if any aberrant read through transcription might overlap genes and contribute to increased antisense reads within the gene. We define an overlapped gene as any gene that has an ADoG associated with it or an opposite strand DoG with any overlap to the gene. We next define a significant overlapped gene as any gene that has significant (FDR <0.05, log2Mean > 4) antisense transcript levels as well as an associated significant (FDR <0.05, log2Mean > 4) ADoG or opposite strand DoG (overlapping the gene). From our significant overlapped genes, we found 17 enriched and 5 depleted with heat shock, and only 4 enriched and no depleted in *tdp-1(ok803)* worms. We did not find any overlapped genes that were significantly enriched for GO terms related to translation (S5 File for list of overlapped genes, S3 File for overlap methods).

### Antisense read through into *eif-3*.*B* in heat shocked worms

**Fig 6:**
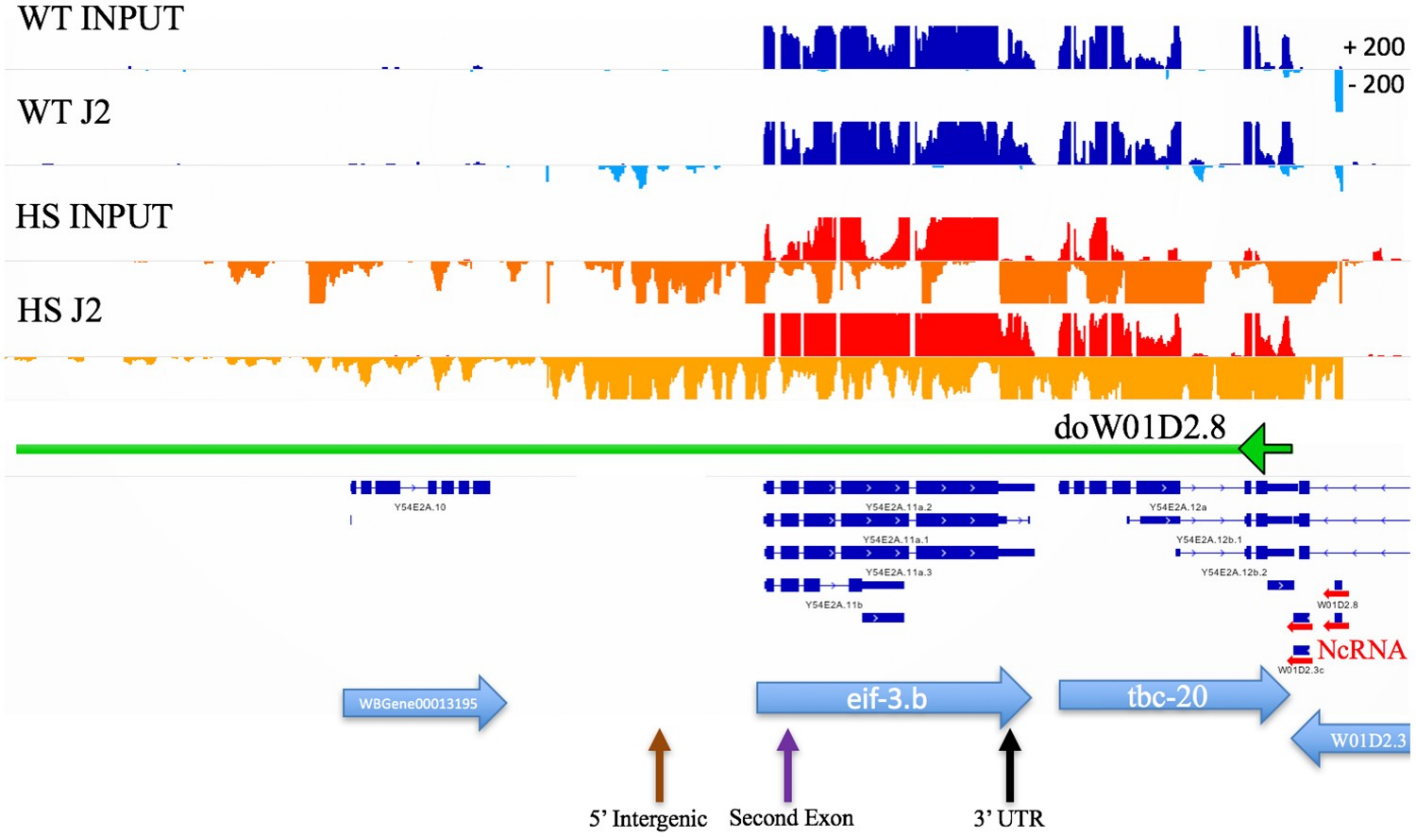
Heat shock induces transcripts antisense to the *eif-3B* locus. IGV view of *eif-3*.B. Normalized tracks with the sense strand on the top part of the histogram and antisense on the bottom with a max read depth of 200 for sense or antisense. Wild type (WT) sense (dark blue), WT antisense (light blue), heat shock (HS) sense (red), heat shock antisense (orange). Horizontal blue arrows indicated genes and gene direction 5’ to 3’. Horizontal red arrows on the right show a cluster of ncRNAs including *W01D2*.*8* and transcription downstream of *W01D2*.*8 (doW01D2*.*8)* into *eif-3*.*B* (green arrow going left). Arrows on the bottom correspond to locations of probes for FISH (brown: 5’ Intergenic, purple: Second exon, black: 3’ UTR).

Visual inspection of DoG transcripts identified one transcript downstream of the ncRNA *W01D2*.*8* (*doW01D2*.*8*) that ran into the gene *eif-3*.*B* on the opposite strand (Fig 6) (Since *doW01D2*.*8* is inside of the gene W01D2.3 on the same strand, annotation of this DoG starts at the end of the W01D2.3). *eif-3*.*B* is an ortholog of human EIF-3.B (eukaryotic translation initiation factor 3 subunit B) and is involved in translation initiation. As the *doW01D2*.*8* transcript was strongly increased by heat shock in both the input and J2 IP pools, we chose to target this transcript to confirm our RNA-seq data. Fluorescent *in situ* hybridization (FISH) was used as this could both demonstrate the accumulation of the *doW01D2*.*8* transcript and determine its cellular and subcellular (i.e., possible colocalization with J2 foci) distribution.

**Fig 7:**
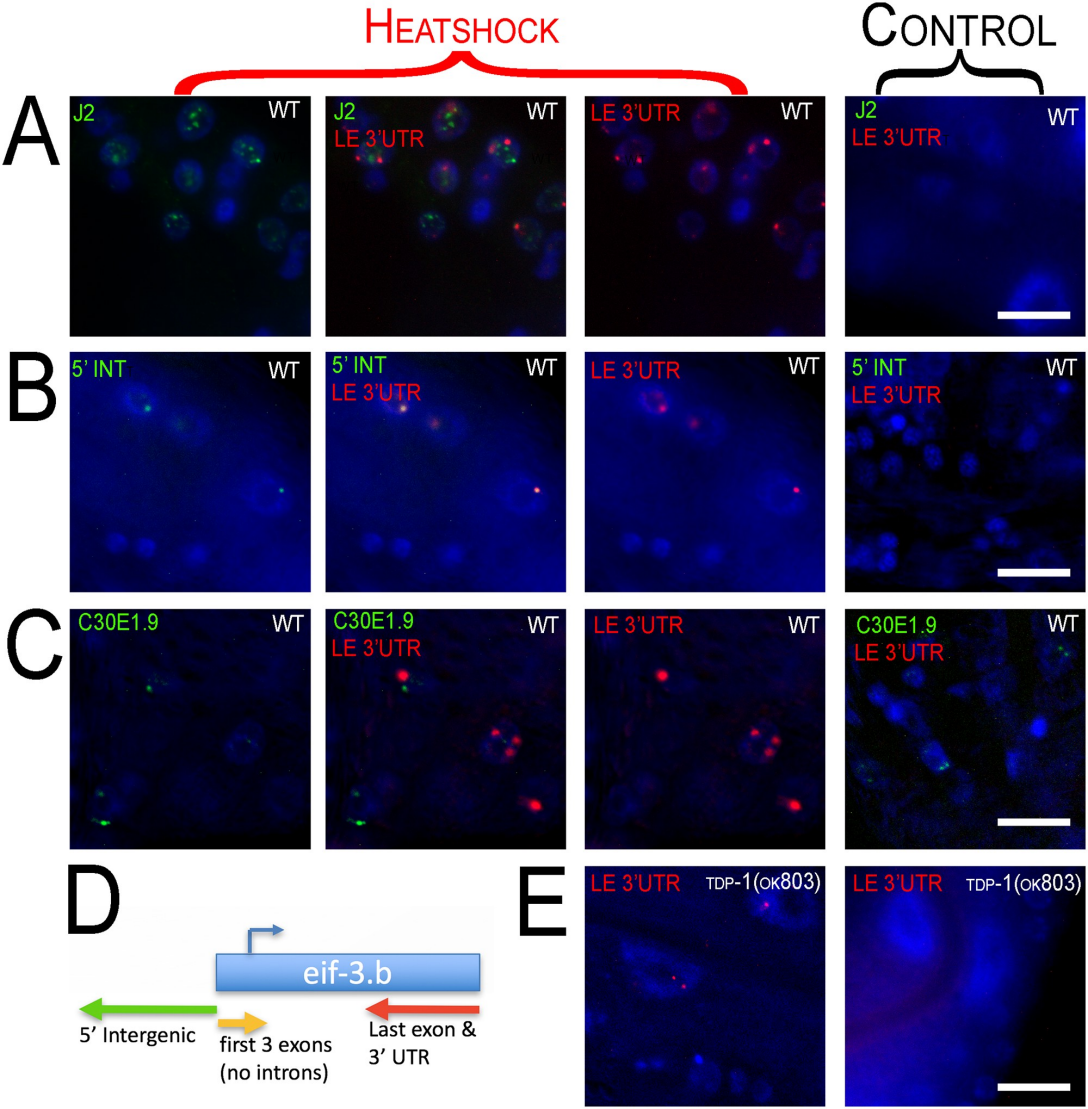
*Fluorescence in situ Hybridization (FISH)* of *eif-3*.*B regions*. 100x oil immersion images of worm hypodermal and neuronal cells. Heat shock panels are in the three columns to the left (merged channel in the middle column). Control panels show exposure from every channel (right column). Row (A) Immunohistochemistry with J2 antibody (green) along with FISH of *doW01D2*.*8* antisense to the last exon and 3’ UTR (LE 3’ UTR) (red) of *eif-3*.*B*. dsRNA and the antisense LE 3’UTR transcript aggregate into nuclear foci with heat shock and do not appear to colocalize. Row (B) FISH of *doW01D2*.*8* in two regions antisense to the 5’ intergenic region (5’ INT) (green) and last exon and 3’ UTR (LE 3’UTR) (red) of *eif-3*.*B*. Row (C) FISH of *doW01D2*.*8* antisense to the last exon and 3’ UTR (LE 3’UTR) (red) of *eif-3*.*B* and sense probe of ncRNA C3DE1.9 (green). Probing of C3DE1.9 is not affected by heat shock and C3DE1.9 is not induced by heat shock. C3DE1.9 and LE 3’UTR show no overlap. (D) Diagram of *eif-3*.*b* gene with FISH probe locations and orientation. (E) Heat shock of *tdp-1(ok803)* induces nuclear foci from probes antisense to the last exon and 3’ UTR (LE 3’UTR) of *eif-3*.*B* (left panel) and is not visible with no heat shock (right).

Three strand-specific fish probes at the 5’ intergenic region (5’ INT) (antisense), first 3 exons (sense), and the last exon along with the 3’ UTR (LE 3’UTR) (antisense) of *eif-3*.*B* (Fig 7D) were designed (list of probes in S1 File). First, we performed immunohistochemistry with the J2 antibody along with FISH for antisense transcripts that contain the last exon and 3’ UTR (see Material and methods) (Fig 7A). We find that *doW01D2*.*8* is transcribed in this region with heat shock and commonly forms two foci per nucleus, but does not colocalize with dsRNA foci.

The 5' and 3’ doW01D2.8 probes do strongly colocalize in the nuclear foci (Fig 7B), consistent with a single transcript spanning this region. Unfortunately, when probing for the 5’ intergenic region (antisense) and first three exons (sense), the sense probe was undetectable in young adults. However, in embryos the sense probe was detectable and we did not find the sense probes colocalizing to the antisense foci (S12 Fig). To inquire if the *eif-3B* antisense foci were a general site of transcript accumulation, we probed for *C30E1*.*9*, a long ncRNA that is highly expressed, forms nuclear foci, but is not induced in heat shock. We observed that this transcript does not overlap with the *eif-3B* antisense foci (Fig 7C). Lastly, we wanted to see if deletion of *tdp-1*, which does not lead to accumulation of *eif-3B* antisense transcripts, would alter heat shock induced accumulation of these transcripts. We found that the *tdp-1* deletion did not alter the formation of *eif-3B* antisense transcripts (Fig 7E).

## Discussion

In our previous study [21] we established that in *C*. *elegans* deletion of *tdp-1* induces nuclear dsRNA foci. Here, we show that heat shock also induces nuclear dsRNA foci that partially overlap with HSF-1 nuclear stress granules. A loss of function mutation in *hsf-1* does not block the formation of heat shock induced dsRNA foci, although because this is a hypomorphic mutation we cannot exclude the possibility that HSF-1 plays some role in the formation of heat shock induced dsRNA foci. After heat shock, we find a general increase in the amount of dsRNA and expression levels of transcripts with dsRNA structure, assayed using the dsRNA-specific monoclonal antibody J2. The dsRNA transcripts recovered by J2 immunoprecipitation after heat shock partially overlap with J2 transcripts previously identified in *C*. *elegans* worms deleted for *tdp-1*. This result suggests that while heat shock does not directly mimic the effects of loss of *tdp-1*, these two conditions likely share some overlapping biological processes. In addition, we find that heat shock induces accumulation of novel downstream of gene transcripts. To our knowledge this is the first time heat shock has been shown to lead to the accumulation of these abnormal transcripts in an *in vivo* model.

dsRNA can form intrastrand or interstrand base-pairing. Our data suggest that both types of dsRNA may be contributing to the dsRNA pool induced by heat shock. We find that novel downstream of gene transcripts are enriched in the J2 IP pool. These novel transcripts are enriched in inverted repeat sequences, which may be contributing to the formation of intrastrand (hairpin) dsRNA. Downstream of gene transcripts also have the potential to generate transcripts antisense to neighboring genes on the other strand. This has been reported in the heat shock study by Vilborg et al [16], and we have noted similar examples in our data (see Fig 7). Using our new Dogcatcher algorithm, we have also documented novel transcripts originating in intergenic regions, which also have the potential to generate antisense transcripts. Indeed, we observe that antisense transcripts are enriched in the J2 IP, supporting the formation of interstrand dsRNA. We note that the J2 antibody immunoprecipitation protocol used in our study will recover transcripts that have only partial (at least 40 nucleotides) dsRNA structure, thus it is feasible that some transcriptional regions we recover after J2 IP are single stranded extensions of double stranded regions.

The accumulation of dsRNA transcripts after heat shock could be the result of altered RNA production and changes in RNA stability or turnover. Further studies will be required to definitively determine the relative contribution of these cellular processes. Published studies demonstrate that loci susceptible to heat shock induced downstream of gene transcription are marked by open chromatin before heat shock [16] and are depleted of the transcriptional termination factor CPSF-73 after heat shock [37]. These results suggest that altered transcriptional processing itself leads to the altered transcript accumulation after heat shock. However, the significant overlap of transcripts enriched in the J2 pool resulting from heat shock and from deletion of the *tdp-1* gene suggest that changes in RNA stability may be also contributing to transcript accumulation. TDP-1 is orthologous to mammalian TDP-43, and we have previously shown that human TDP-43 can act as an RNA chaperone in an *in vitro* assay [21]. Conceivably, heat shock could inhibit the function of TDP-1 or other similar RNA binding proteins, leading to the formation of more dsRNA structure in existing transcripts.

We employed fluorescence in situ hybridization (FISH) to confirm heat shock induced expression of DoG and antisense transcripts in the *eif-3*.*B* region, and to examine their subcellular localization. These novel transcripts were found in nuclear foci that did not overlap with the J2 dsRNA foci, and were typically limited to two spots in each nuclei. This two foci distribution is very similar to the FISH characterization of DoG transcripts described by Vilborg et al, and strongly suggest that the *eif-3*.*B* loci transcripts are associated in *cis* with their site of production. These antisense transcripts clearly did not contribute to the foci detected by J2 immunostaining, and may reflect a general dysregulation of transcription at the *eif-3*.*B* locus. Identification of the dsRNA species present in the J2 foci induced by heat shock may require development of a protocol to purify these RNA granules, as we have identified thousands of transcripts enriched in the J2 pool, and have no additional insight as to which ones might be found specifically in the J2 foci.

A critical issue is whether the accumulation of novel transcripts and dsRNA after heat shock have a biological function. By characterizing transcriptional changes induced by a variety of stresses, Vilborg et al concluded that transcriptional read through was not a random failure, and suggested it might have a functional role in stress responses. We have characterized the accumulation of dsRNA after heat shock, and by gene ontology analysis find that the sense and antisense transcripts in this pool (as well as the J2 IP pool in *tdp-1* deletion mutants) are enriched in genes involved in translation. Given that we find significant J2 IP enrichment of both sense and antisense transcripts from genes related to translation, it is tempting to speculate that the formation of interstrand dsRNA might reduce the translation of these “translation related transcripts”, leading to a down regulation of global translation, a protective event against most cellular stress insults including heat shock. While we have no direct evidence that dsRNA dependent translational downregulation happens after heat shock in *C*. *elegans*, we note that deletion of *tdp-1* has been reported to protect against proteotoxicity and increase lifespan [38]. Translational downregulation would presumably be protective against proteotoxicity, and post developmental knockdown of translation initiation factors strongly increases lifespan in *C*. *elegans* [39].

## Supporting information

S1 Fig Quantification of J2 foci with or without heatshock in N2, *rde-4*, and *sy441* mutants. (TIF)

S2 Fig Schematic of recovery of RNA pools for high throughput sequencing analysis. (TIF)

S3 Fig Comparison of antisense transcripts in worms with heat shock (wild type) vs no heat shock (wild type) from Brunquell 2016. (TIF)

S4 Fig Comparison of heat shock J2 enriched transcripts significant in both sense and antisense. (TIF)

S5 Fig Comparison of *tdp-1(ok803)* J2 enriched transcripts significant in both sense and antisense. (TIF)

S6 Fig Upset plots of all DoGs and DoGs significant with heat shock. (TIF)

S7 Fig Venn Diagram of overlap between significant genes and DoGs. (TIF)

S8 Fig Number of Terminal Inverted Repeats (TIR) overlapping downstream regions. (TIF)

S9 Fig Comparison of DoGs and ADoGs in worms with heat shock (wild type) vs no heat shock (wild type) from Brunquell 2016. (TIF)

S10 Fig Dogcatcher flattening and nomenclature. (TIF)

S11 Fig Dogcatcher additional filtering. (TIF)

S12 Fig Sense and antisense *eif-3B* transcripts do not colocalize. (TIF)

S1 Table Worm strains used in this study. (XLSX)

S2 Table Quantification of coincidence of J2 and HSF-1 foci over time and J2 foci in mutant strains (raw data). (XLSX)

S1 File Probes used in fluorescence in situ hybridization (FISH) of eif-3.B regions. (DOCX)

S2 File List and sequences of adapters used in trimming. (FA)

S3 File Bioinformatic methods. (PDF)

S4 File Hypergeometric Distribution and list of genes or DoGs used in calculation for heat shock and *tdp-1(ok803*). (XLSX)

S5 File List of significant genes, translation associated genes, and overlapped genes. (XLSX)

S6 File Significantly enriched GO terms for heat shock and *tdp-1(ok803)* genes. (XLSX)

S7 File List of significant DoGs, ADoGs, PoGs, APoGs. (XLSX)

S8 File Significantly enriched GO terms for heat shock DoGs. (XLSX)

## References

[1] Lindquist S. The Heat-Shock Response. Annu Rev Biochem. Annual Reviews 4139 El Camino Way, P.O. Box 10139, Palo Alto, CA 94303–0139, USA; 1986;55: 1151–1191. 10.1146/annurev.bi.55.070186.005443 2427013

[2] Gomez-Pastor R, Burchfiel ET, Thiele DJ. Regulation of heat shock transcription factors and their roles in physiology and disease. Nat Rev Mol Cell Biol. Nature Publishing Group; 2018;19: 4–19. 10.1038/nrm.2017.73 28852220 PMC5794010

[3] Brewer-Jensen P, Wilson CB, Abernethy J, Mollison L, Card S, Searles LL. Suppressor of sable [Su(s)] and Wdr82 down-regulate RNA from heat-shock-inducible repetitive elements by a mechanism that involves transcription termination. RNA. 2016;22: 139–54. 10.1261/rna.048819.114 26577379 PMC4691828

[4] Shalgi R, Hurt JA, Lindquist S, Burge CB. Widespread inhibition of posttranscriptional splicing shapes the cellular transcriptome following heat shock. Cell Rep. The Authors; 2014;7: 1362–1370. 10.1016/j.celrep.2014.04.044 24857664

[5] Vilborg A, Passarelli MC, Yario TA, Tycowski KT, Steitz JA. Widespread Inducible Transcription Downstream of Human Genes. Mol Cell. 2015;59: 449–461. 10.1016/j.molcel.2015.06.016 26190259 PMC4530028

[6] Gidalevitz T, Prahlad V, Morimoto RI. The stress of protein misfolding: From single cells to multicellular organisms. Cold Spring Harb Perspect Biol. 2011;3: 1–18. 10.1101/cshperspect.a009704 21536706 PMC3098679

[7] Westwood JT, Clos J, Wu C. Stress-induced oligomerization and chromosomal relocalization of heat-shock factor. Nature. 1991;353: 822–827. 10.1038/353822a0 1944557

[8] Åkerfelt M, Morimoto RI, Sistonen L. Heat shock factors: Integrators of cell stress, development and lifespan [Internet]. Nature Reviews Molecular Cell Biology. NIH Public Access; 2010 pp. 545–555. 10.1038/nrm2938 20628411 PMC3402356

[9] Lindquist S. Regulation of protein synthesis during heat shock. Nature. Nature Publishing Group; 1981;293: 311–314. 10.1038/293311a0 6792546

[10] Shalgi R, Hurt JA, Krykbaeva I, Taipale M, Lindquist S, Burge CB. Widespread Regulation of Translation by Elongation Pausing in Heat Shock. Mol Cell. 2013;49: 439–452. 10.1016/j.molcel.2012.11.028 23290915 PMC3570722

[11] Sonenberg N, Hinnebusch AG. Regulation of Translation Initiation in Eukaryotes: Mechanisms and Biological Targets [Internet]. Cell. 2009 pp. 731–745. 10.1016/j.cell.2009.01.042 19239892 PMC3610329

[12] Smith HL, Li W, Cheetham ME. Molecular chaperones and neuronal proteostasis. Semin Cell Dev Biol. 2015;40: 142–152. 10.1016/j.semcdb.2015.03.003 25770416 PMC4471145

[13] Jolly C, Metz A, Govin J, Vigneron M, Turner BM, Khochbin S, et al Stress-induced transcription of satellite III repeats. J Cell Biol. 2004;164: 25–33. 10.1083/jcb.200306104 14699086 PMC2171959

[14] Morton EA, Lamitina T. Caenorhabditis elegans HSF-1 is an essential nuclear protein that forms stress granule-like structures following heat shock. Aging Cell. 2013;12: 112–120. 10.1111/acel.12024 23107491 PMC3552056

[15] Proudfoot NJ. Transcriptional termination in mammals: Stopping the RNA polymerase II juggernaut [Internet]. Science. American Association for the Advancement of Science; 2016 p. aad9926. 10.1126/science.aad9926 27284201 PMC5144996

[16] Vilborg A, Sabath N, Wiesel Y, Nathans J, Levy-Adam F, Yario TA, et al Comparative analysis reveals genomic features of stress-induced transcriptional readthrough. Proc Natl Acad Sci. 2017;114: E8362–E8371. 10.1073/pnas.1711120114 28928151 PMC5635911

[17] Rutkowski AJ, Erhard F, L’Hernault A, Bonfert T, Schilhabel M, Crump C, et al Widespread disruption of host transcription termination in HSV-1 infection. Nat Commun. 2015;6 10.1038/ncomms8126 25989971 PMC4441252

[18] Grosso AR, Leite AP, Carvalho S, Matos MR, Martins FB, Vítor AC, et al Pervasive transcription read-through promotes aberrant expression of oncogenes and RNA chimeras in renal carcinoma. Elife. 2015;4: 1–16. 10.7554/eLife.09214 26575290 PMC4744188

[19] Muniz L, Deb MK, Aguirrebengoa M, Lazorthes S, Trouche D, Nicolas E. Control of Gene Expression in Senescence through Transcriptional Read-Through of Convergent Protein-Coding Genes. Cell Rep. ElsevierCompany.; 2017;21: 2433–2446. 10.1016/j.celrep.2017.11.006 29186682

[20] Wight M, Werner A. Europe PMC Funders Group The functions of natural antisense transcripts. 2015; 91–101. 10.1042/bse0540091 23829529 PMC4284957

[21] Saldi TK, Ash PE, Wilson G, Gonzales P, Garrido-Lecca A, Roberts CM, et al TDP-1, the Caenorhabditis elegans ortholog of TDP-43, limits the accumulation of double-stranded RNA. EMBO J. 2014;33: 2947–66. 10.15252/embj.201488740 25391662 PMC4282642

[22] Brenner S. The genetics of Caenorhabditis elegans. Genetics. 1974;77: 71–94. 4366476 10.1093/genetics/77.1.71 PMC1213120

[23] Porta-de-la-Riva M, Fontrodona L, Villanueva A, Cerón J. Basic Caenorhabditis elegans methods: synchronization and observation. J Vis Exp. MyJoVE Corporation; 2012; e4019 10.3791/4019 22710399 PMC3607348

[24] Orjalo A, Johansson HE, Ruth JL. Stellaris fluorescence in situ hybridization (FISH) probes: A powerful tool for mRNA detection. Nat Methods. 2011;8: i–ii. 10.1038/nmeth.f.349

[25] Thorvaldsdóttir H, Robinson JT, Mesirov JP. Integrative Genomics Viewer (IGV): high-performance genomics data visualization and exploration. Brief Bioinform. 2013;14: 178–92. 10.1093/bib/bbs017 22517427 PMC3603213

[26] Andrews S. FastQC: A quality control tool for high throughput sequence data. http://www.bioinformatics.babraham.ac.uk/projects/fastqc/. 2017; 1.

[27] Bolger AM, Lohse M, Usadel B. Trimmomatic: A flexible trimmer for Illumina sequence data. Bioinformatics. Oxford University Press; 2014;30: 2114–2120. 10.1093/bioinformatics/btu170 24695404 PMC4103590

[28] Dobin A, Gingeras TR. Mapping RNA-seq Reads with STAR [Internet]. Current protocols in bioinformatics. NIH Public Access; 2015 p. 11.14.1–11.14.19. 10.1002/0471250953.bi1114s51 26334920 PMC4631051

[29] Liao Y, Smyth GK, Shi W. FeatureCounts: An efficient general purpose program for assigning sequence reads to genomic features. Bioinformatics. 2014;30: 923–930. 10.1093/bioinformatics/btt656 24227677

[30] Love MI, Huber W, Anders S. Moderated estimation of fold change and dispersion for RNA-seq data with DESeq2. Genome Biol. 2014;15: 1–21. 10.1186/s13059-014-0550-8 25516281 PMC4302049

[31] Morton EA, Lamitina T. Caenorhabditis elegans HSF-1 is an essential nuclear protein that forms stress granule-like structures following heat shock. Aging Cell. NIH Public Access; 2013;12: 112–20. 10.1111/acel.12024 23107491 PMC3552056

[32] Parker GS, Eckert DM, Bass BL. RDE-4 preferentially binds long dsRNA and its dimerization is necessary for cleavage of dsRNA to siRNA. RNA. Cold Spring Harbor Laboratory Press; 2006;12: 807–18. 10.1261/rna.2338706 16603715 PMC1440910

[33] Kaneko H, Dridi S, Tarallo V, Gelfand BD, Fowler BJ, Cho WG, et al DICER1 deficit induces Alu RNA toxicity in age-related macular degeneration. Nature. 2011;471: 325–332. 10.1038/nature09830 21297615 PMC3077055

[34] Daume M, Uhl M, Backofen R, Randau L. RIP-seq suggests translational regulation by L7Ae in Archaea. MBio. American Society for Microbiology; 2017;8: e00730–17. 10.1128/mBio.00730-17 28765217 PMC5539422

[35] Brunquell J, Morris S, Lu Y, Cheng F, Westerheide SD. The genome-wide role of HSF-1 in the regulation of gene expression in Caenorhabditis elegans. BMC Genomics. BMC Genomics; 2016;17: 1–18. 27496166 10.1186/s12864-016-2837-5 PMC4975890

[36] Klopfenstein D V., Zhang L, Pedersen BS, Ramírez F, Warwick Vesztrocy A, Naldi A, et al GOATOOLS: A Python library for Gene Ontology analyses. Sci Rep. Nature Publishing Group; 2018;8: 10872 10.1038/s41598-018-28948-z 30022098 PMC6052049

[37] Cardiello JF, Goodrich JA, Kugel JF. Heat shock causes a reversible increase in RNA polymerase II occupancy downstream of mRNA genes consistent with a global loss in transcriptional termination. Mol Cell Biol. American Society for Microbiology Journals; 2018;38: MCB.00181-18. 10.1128/MCB.00181-18 29967245 PMC6113597

[38] Zhang T, Hwang HY, Hao H, Talbot C, Wang J. Caenorhabditis elegans RNA-processing protein TDP-1 regulates protein homeostasis and life span. J Biol Chem. 2012;287: 8371–8382. 10.1074/jbc.M111.311977 22232551 PMC3318719

[39] Curran SP, Ruvkun G. Lifespan regulation by evolutionarily conserved genes essential for viability. PLoS Genet. 2007;3: e56 10.1371/journal.pgen.0030056 17411345 PMC1847696

