# Supplementary figures and images for: Heat shock in C. elegans induces downstream of gene transcription and accumulation of double-stranded RNA
Source: PLoS One. 2019 Apr 8;14(4):e0206715. doi: 10.1371/journal.pone.0206715 (PMC6453478; doi:10.1371/journal.pone.0206715)

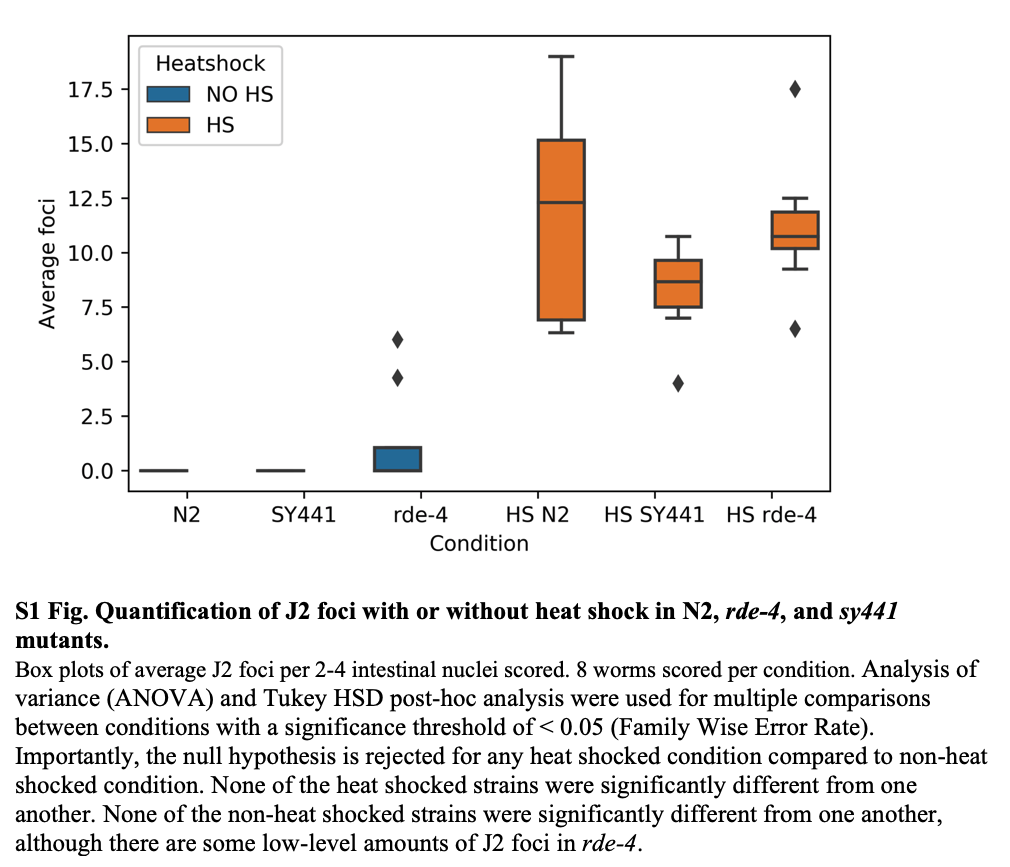

Supplement: S1 Fig — (TIF) [file pone.0206715.s001.tif]

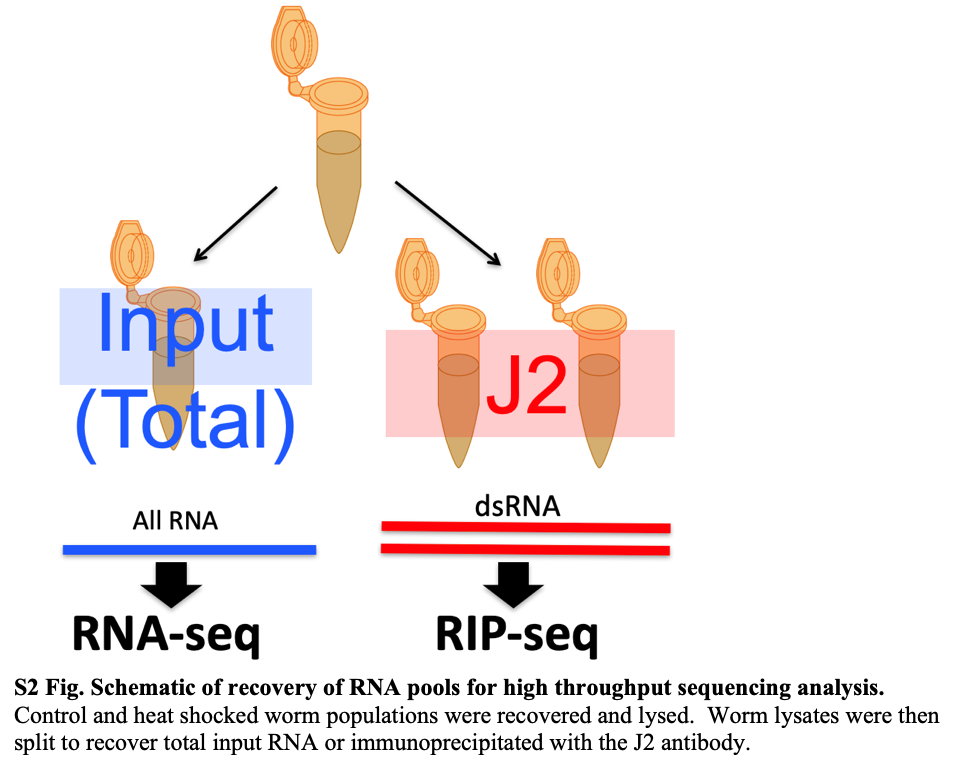

Supplement: S2 Fig — (TIF) [file pone.0206715.s002.tif]

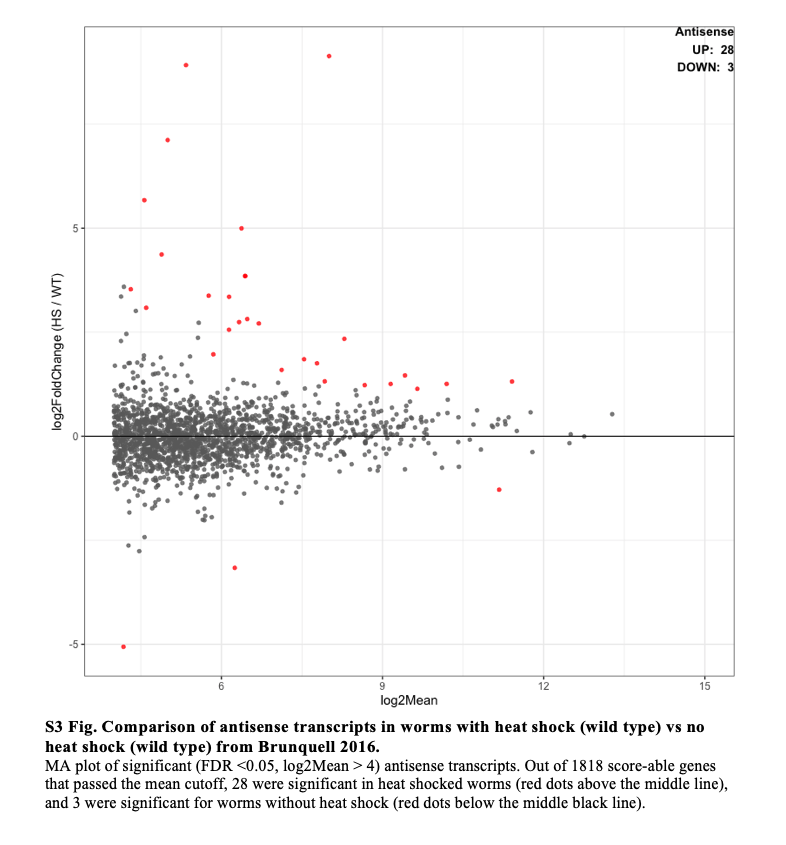

Supplement: S3 Fig — (TIF) [file pone.0206715.s003.tif]

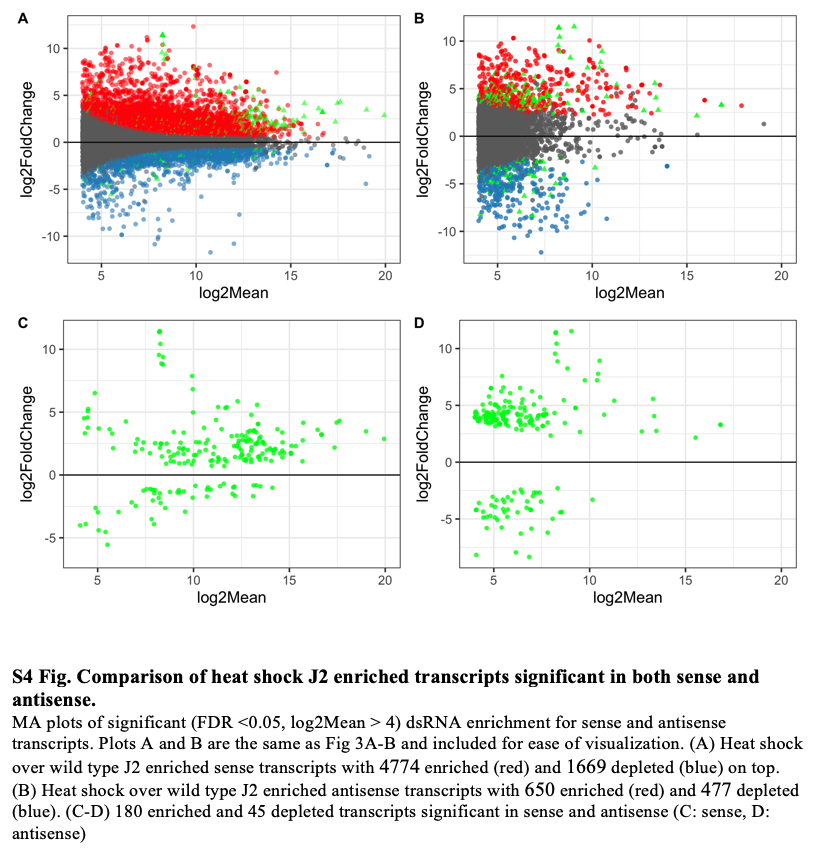

Supplement: S4 Fig — (TIF) [file pone.0206715.s004.tif]

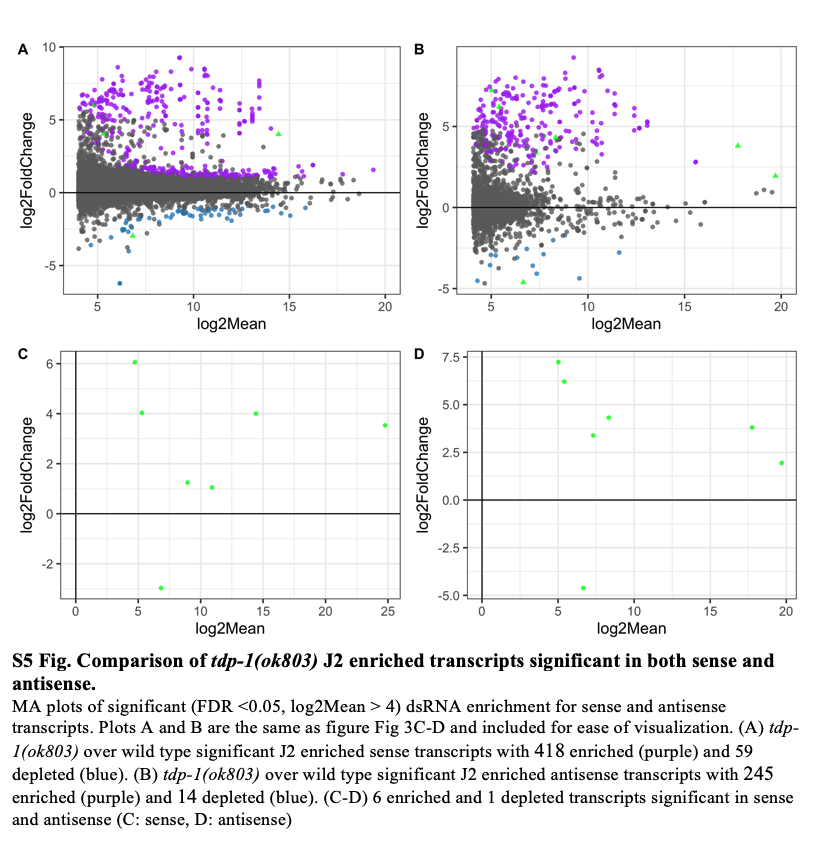

Supplement: S5 Fig — (TIF) [file pone.0206715.s005.tif]

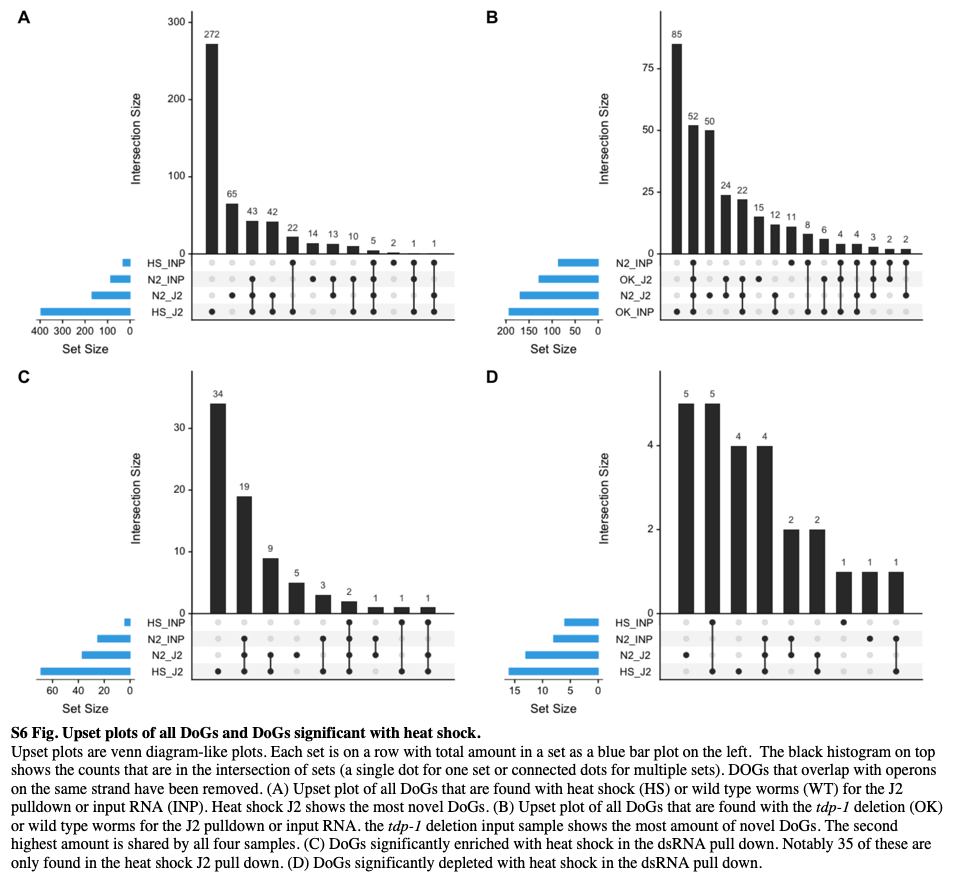

Supplement: S6 Fig — (TIF) [file pone.0206715.s006.tif]

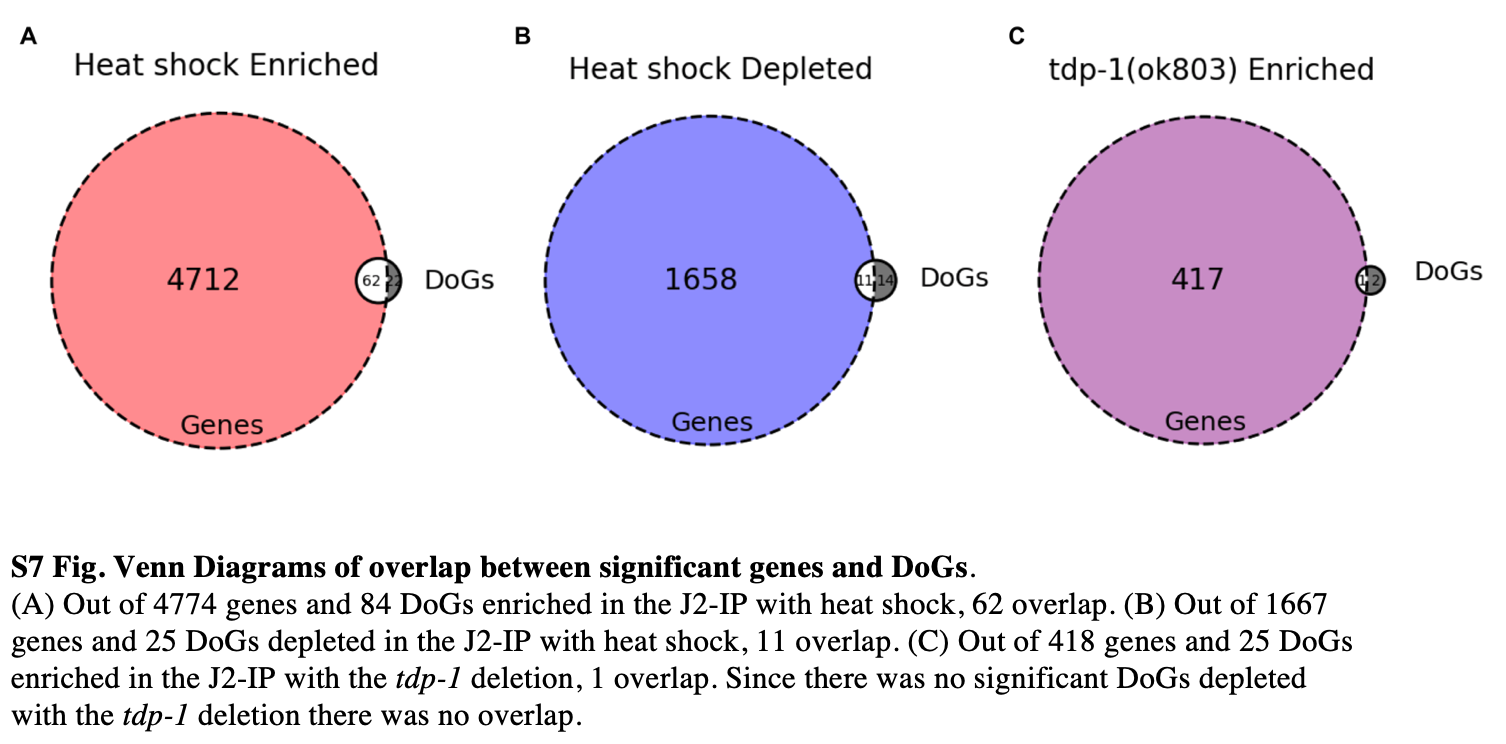

Supplement: S7 Fig — (TIF) [file pone.0206715.s007.tif]

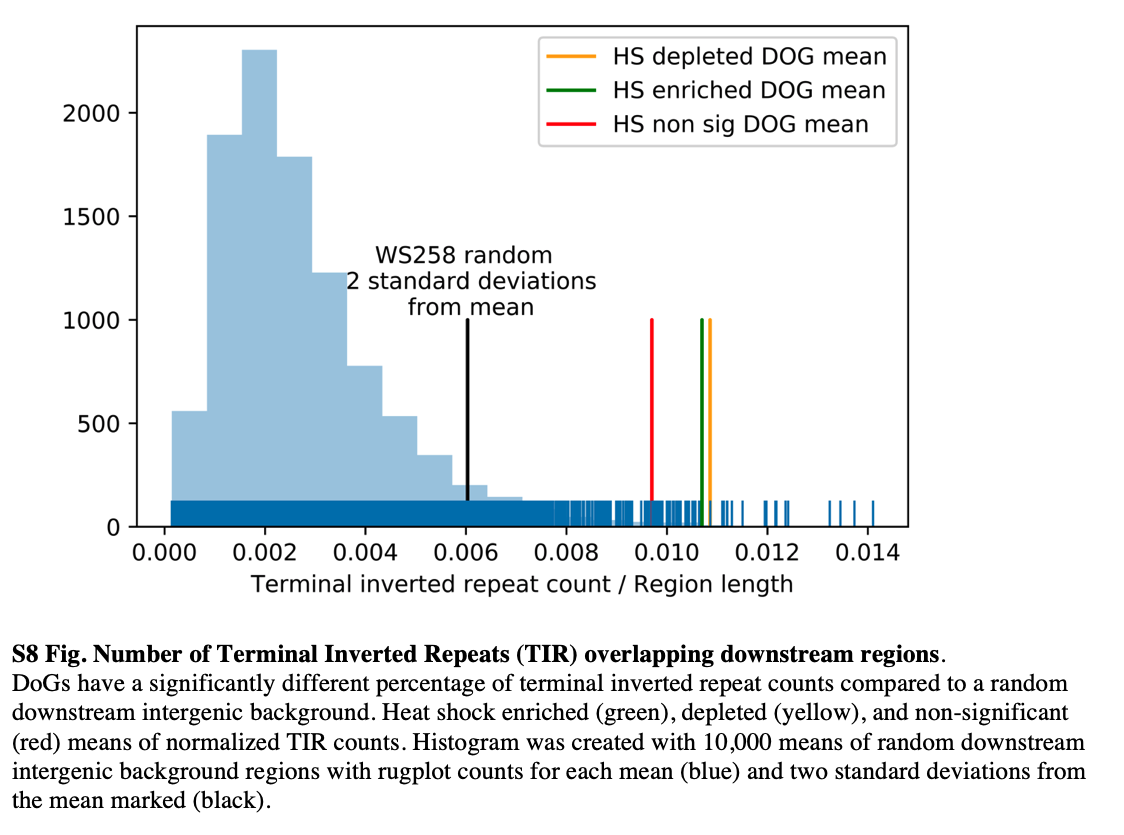

Supplement: S8 Fig — (TIF) [file pone.0206715.s008.tif]

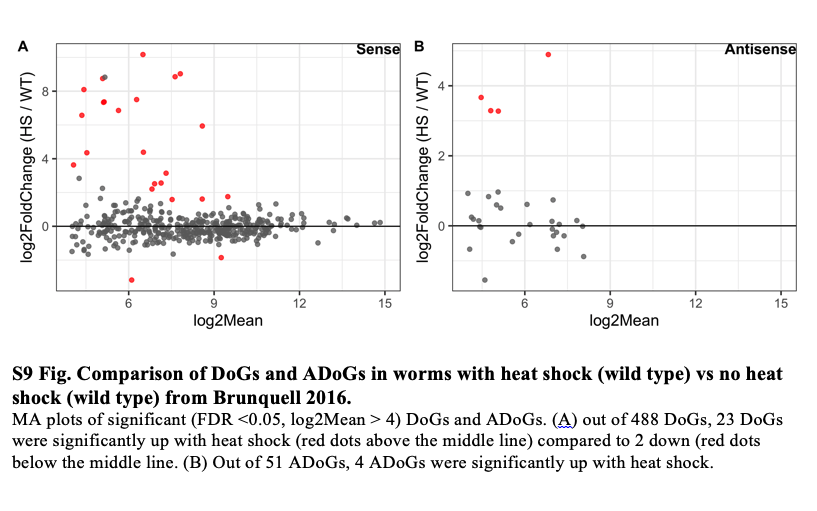

Supplement: S9 Fig — (TIF) [file pone.0206715.s009.tif]

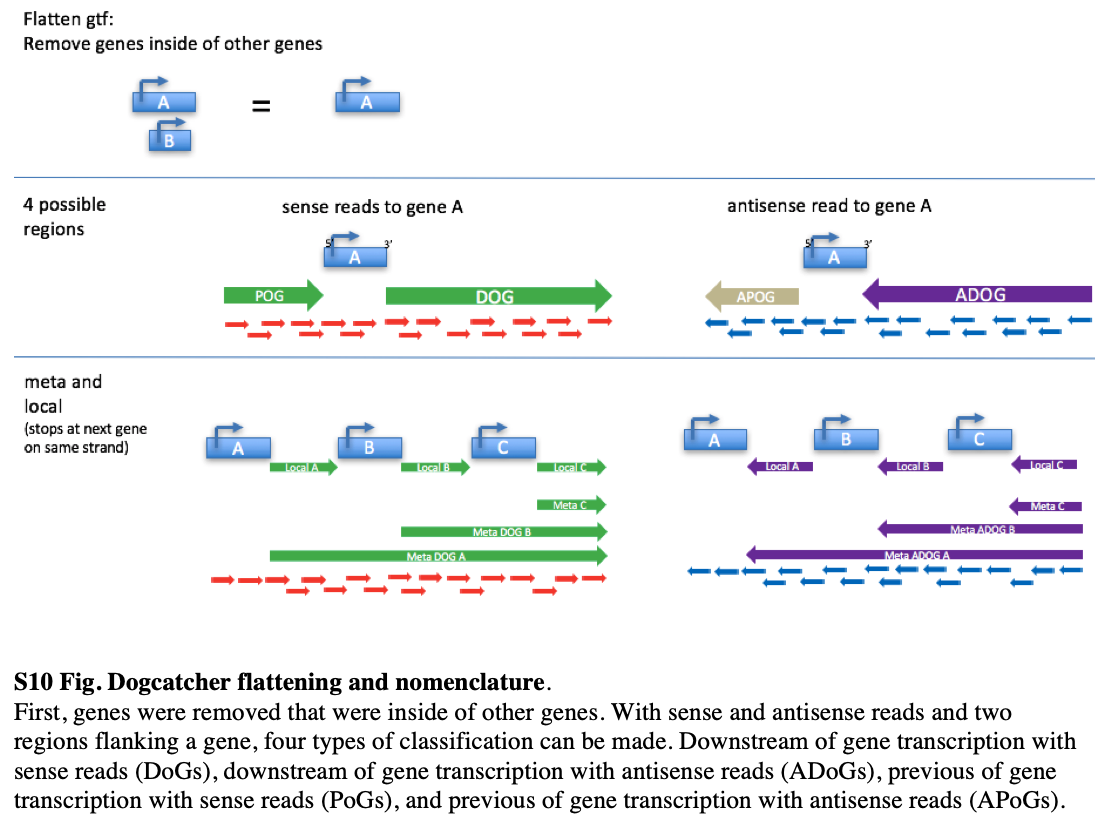

Supplement: S10 Fig — (TIF) [file pone.0206715.s010.tif]

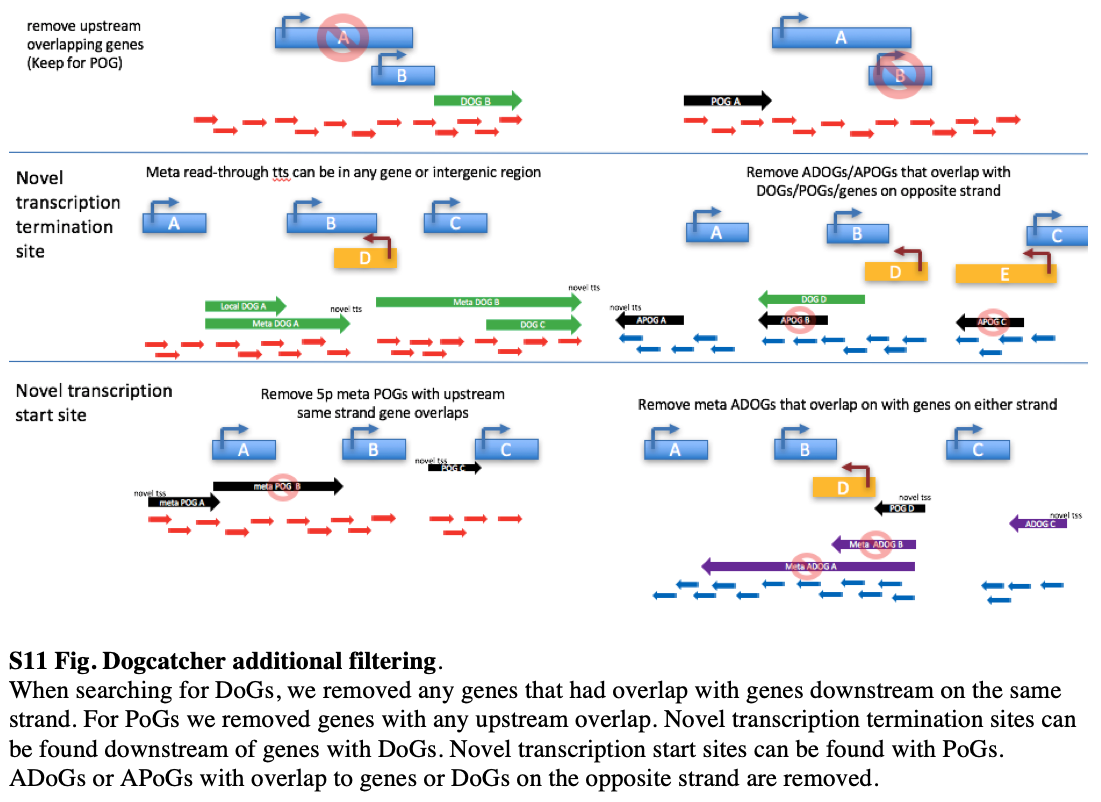

Supplement: S11 Fig — (TIF) [file pone.0206715.s011.tif]

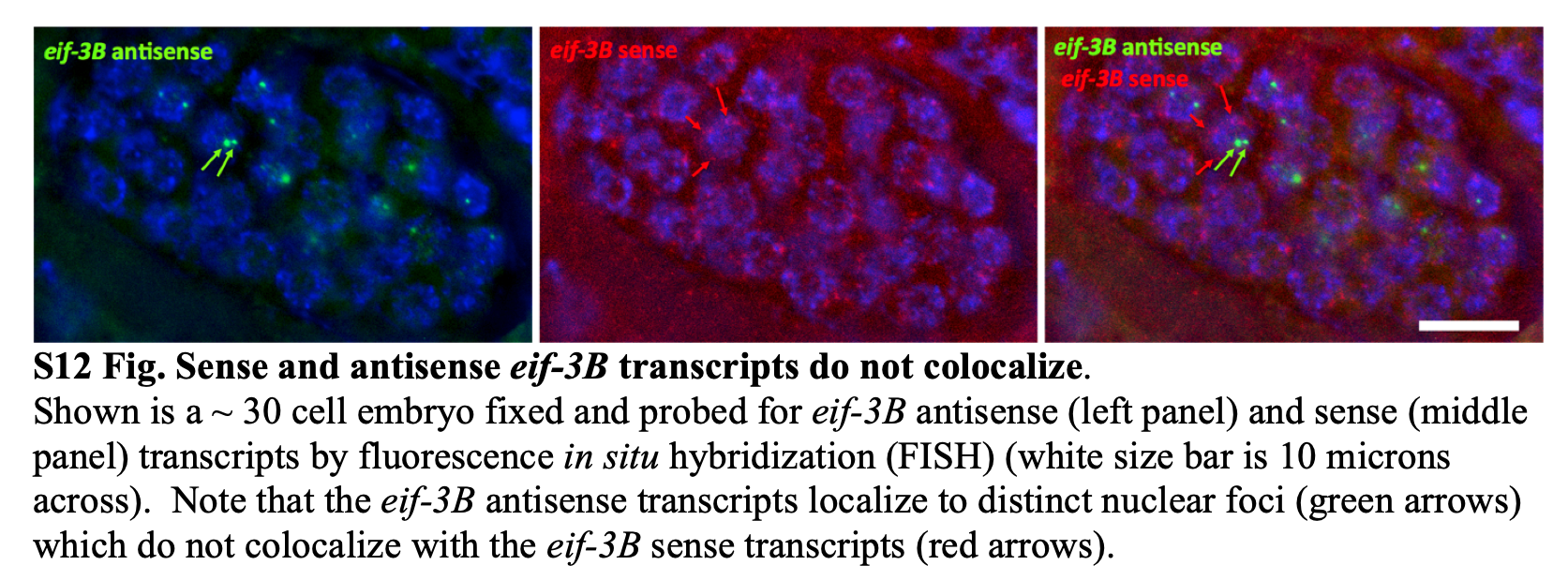

Supplement: S12 Fig — (TIF) [file pone.0206715.s012.tif]
