# Supplementary material for: Heat shock in C. elegans induces downstream of gene transcription and accumulation of double-stranded RNA
Source: PLoS One. 2019 Apr 8;14(4):e0206715. doi: 10.1371/journal.pone.0206715 (PMC6453478; doi:10.1371/journal.pone.0206715)
Supplement: S1 File — (DOCX) [file pone.0206715.s015.docx]

**File S1 -** probes used for fluorescent in situ hybridization (FISH) studies

**Last_exon_3putr**

**Reverse Compliment Quasar 570** last_exon_3putr_eif3-b

tcacttctgtttatttggaaaaaatctgaaacatcaactaattcatcaacattttatcggcaaaaaccaccagaaaacacggagagacgggagcaacaagagatattaaagtggatagagagaaacaattaaataatattagggccgcccaaaagaagcttgaaaaaaccttctccattcaggcatcataaagttaaatttgccatagaaaaaggcgttgttcagaaaatgacgacatgactgggaaaatgaagaggaaaacgaaaacgagtacatggtggaaccaccaaaaagaggggacgccgctccaagtcttcgagaaatcaggaagaaaaagcagcgcataaaaaaggggaaaaaccgggaaatcaaataactaaTTAGTCTCTCATCTCCTCCTCCGTCAGAGGGGCTTGTGTTTTGCTTGTGCTCAGCGCAATGGTAATCTCCTCATCCACAAATTCGTCCTCGTCGAGTTGGGCTTCTGTGTCCACGCCGTTTCGAAGAGAGATTCTCTCGTCACGGGTGGCGTCGAGTTGCTCGCGATTCCGGCTTCGAATAATATCGAAGGCAGCCATGATCTTGCGTCGCTTCTCAACAACCTCCTGACTGGCTCTGCACTTTTCGTCGTCATCCTGCTTGATGAACTTGGCGGCGGTCTTCTTCAAGTTCTTCTTGATCTCACGCTGCTTCTGTTCGCTCAACTTCACCGGCGGCCGTGGTCTCCACTTGAATTGGGCGAGCCGGTCGAGATTCTTTCTGCACAGTTCTCTTCCCTGGAATGTGAAGATTCGGTAACCCAAATCGGCTCCGGCTCTTCCTCCCAACGTTGAGCATGTCACAAAGTATCTTCCAGTTGGATCCCAATATCCCTTATTGAAGAGTGGATGTTCTATAACATTAGTTCTCTTGGCCTCCGACAAAGAAGTATCAATGAAATAGACGTTTCCTCCAGCTGACACCTTGGCGAGAACTGCAAGCCATCCTCCTTTCGGCGCGAATTGAACCTCATTGAAGTGAACTCCAGCGTCCAACTTGCTCACAAGCTTCGGTGCGTGGCTGTTAGCCTCGATCTTGTAGACTTGTGGAGTGGCCTTAGCGGTGTTTCCCACGAGCACACAGAACTTATCGCCTTCTGGATCCCAATCGAAATGGATGAATGGCTCCGAAAGTGGGAGATTCATAAGGGAAACGTCCTTCTTGTCAATCTCGAAGATGTCCACGTGGTATTGGCATCCTCC

| **PROBE #** | **PROBE (5'-> 3')** | **PROBE POSITION *** |
| --- | --- | --- |

|  | 2 | ggtttttgccgataaaatgttg | 47 |
| --- | --- | --- | --- |
|  | 3 | tctctatccactttaatatctc | 100 |
|  | 6 | aactttatgatgcctgaatgga | 174 |
|  | 7 | aacgcctttttctatggcaaat | 198 |
|  | 11 | ttttttatgcgctgctttttct | 330 |
|  | 13 | gaggaggagatgagagactaat | 380 |
|  | 14 | gagcacaagcaaaacacaagcc | 411 |
|  | 15 | ggatgaggagattaccattgcg | 435 |
|  | 17 | agagaatctctcttcgaaacg | 496 |
|  | 18 | gatattattcgaagccggaatc | 545 |
|  | 19 | tgagaagcgacgcaagatcatg | 576 |
|  | 20 | aggatgacgacgaaaagtgcag | 616 |
|  | 22 | ggtgaagttgagcgaacagaag | 690 |
|  | 23 | actgtgcagaaagaatctcgac | 747 |
|  | 24 | aatcttcacattccagggaaga | 771 |
|  | 25 | atgctcaacgttgggaggaaga | 816 |
|  | 31 | cacttcaatgaggttcaattcg | 998 |
|  | 32 | gaagcttgtgagcaagttggac | 1029 |
|  | 33 | aagtctacaagatcgaggctaa | 1063 |
|  | 34 | agaaggcgataagttctgtgtg | 1116 |
|  | 35 | attcatccatttcgattgggat | 1140 |
|  | 36 | aaggacgtttcccttatgaatc | 1178 |
|  | 37 | gtggacatcttcgagattgaca | 1202 |

**5' Intergenic antisense**

**5' to 3'**

**1241 bp**

Earlier 5p intergenic

>II:14792748..14794113
cacatttgtaattgttcaatgaatctgtatattatttagtgtaaactaaaaattatttga
tgaacacacatgataaacttttagatagcacttatgtacatatacatgagaaatccggtg
cttagatatgaactcctttttttaagcataggtttttaggttacctgttttagcgagttt
gaattaatcgtggcaaaactcacagtcgtcaataaaatgaacatatataaagtattcaaa
catacaaagcaacaaaactaattgtaaataaacaaaattaaaaatattttcgatttatta
aaagaacaaaacattattgcgctcaccttgtttgaatcatgtacgatgcattatccgaaa
cggcgagcctgtagcacaatagtgataacacagggttcctcttaaccaaagccgtcggat
ccgaagatctactggcgtttgatcgagttgcccgcctttttcctcacggactagatctct
accccacaatgggattaaacaagaccgaccctagacaatcctagagtcagcttaatctgc
cagcagaatccactgacccttgggtggccgtcccgtttttcaagatttctagggatttgc
gacctttctcacgtcacgtgcgttacccagcaccggccaagtgatgcaagaaagggcttg
gaggtccacatccagtgagtgcttagctacagctaaacgaccactggaacaggaagtccc
cggtgatgatcgtattgaactacacaactttattgtagtaaactacactaccggttatta
tatccagacatagtctcccgatgaaatactctcacttgttcaagtgtgaaacccgaacct
tgctcttgtacagttgataaaactaatgtaactcaaaatatgaaacggcccaagactatg
aaagatattttaagtttccaagttctttcttaaattccaaaacttctttgattcaacaac
tttctactaaaacgctcatagcaaatcatctgcaaggacgaatccattgagaataatgta
tactaatcatcatgttatagtaaaaattgcgtgatttgtacagtaaaaggtcagcagcat
tgttgtgaggactacccccttggattgttgcttgctctttgattgttgagcgggcatcga
ctggagctcggtatcgattgtgctagccaaataaaaatcgattagaggtgtggcatttga
agaacatcaaatttctatagatacgaaagtgtctatatgtatcaagtagcagactaaaaa
aattccaaaaaaaatcgcctaccccacctctattttcgattttaaataaccagtcattcg
attgagcgcgcaattgagtgccgctaggtcatcaaacagggccg

**Reverse compliment**

cggccctgtttgatgacctagcggcactcaattgcgcgctcaatcgaatgactggttatttaaaatcgaaaatagaggtggggtaggcgattttttttggaatttttttagtctgctacttgatacatatagacactttcgtatctatagaaatttgatgttcttcaaatgccacacctctaatcgatttttatttggctagcacaatcgataccgagctccagtcgatgcccgctcaacaatcaaagagcaagcaacaatccaagggggtagtcctcacaacaatgctgctgaccttttactgtacaaatcacgcaatttttactataacatgatgattagtatacattattctcaatggattcgtccttgcagatgatttgctatgagcgttttagtagaaagttgttgaatcaaagaagttttggaatttaagaaagaacttggaaacttaaaatatctttcatagtcttgggccgtttcatattttgagttacattagttttatcaactgtacaagagcaaggttcgggtttcacacttgaacaagtgagagtatttcatcgggagactatgtctggatataataaccggtagtgtagtttactacaataaagttgtgtagttcaatacgatcatcaccggggacttcctgttccagtggtcgtttagctgtagctaagcactcactggatgtggacctccaagccctttcttgcatcacttggccggtgctgggtaacgcacgtgacgtgagaaaggtcgcaaatccctagaaatcttgaaaaacgggacggccacccaagggtcagtggattctgctggcagattaagctgactctaggattgtctagggtcggtcttgtttaatcccattgtggggtagagatctagtccgtgaggaaaaaggcgggcaactcgatcaaacgccagtagatcttcggatccgacggctttggttaagaggaaccctgtgttatcactattgtgctacaggctcgccgtttcggataatgcatcgtacatgattcaaacaaggtgagcgcaataatgttttgttcttttaataaatcgaaaatatttttaattttgtttatttacaattagttttgttgctttgtatgtttgaatactttatatatgttcattttattgacgactgtgagttttgccacgattaattcaaactcgctaaaacaggtaacctaaaaacctatgcttaaaaaaaggagttcatatctaagcaccggatttctcatgtatatgtacataagtgctatctaaaagtttatcatgtgtgttcatcaaataatttttagtttacactaaataatatacagattcattgaacaattacaaatgtg

**1241 bp**

>II:14794113..14795354
tcatcgcaaaaacatagcttgacaaaatgctgctgtgttaccgtactaatcatggagaac
caagaatcatcaatcatgaaaaagcaagacgatccgcagaagaataaaaaagaactgcta
aaaatcatttgtggttttgcctttaacttttcgttttatacaagctatcccttccgtcga
agctggcagattttgtaccggcaatactctcactttttccaatgtaaagcccaaaccttt
ctgttgcacagtcgacaaaactcatctaactcagaatatgaaacgacctaaccctgtcag
agatattttaagttttcaagttctttcttaaaatccaaaacttctttgattcaacaactt
tttactgaaacactcatagtccatactctgcaaggacgaatccaaagagaataatgtaaa
ctaatcatcatgttacagtaaaactaacaaaaacacaactccgtatccagacatagtctt
ccgatgcaatacactcactttttccaatgtaaagcccaaacctttctgttgcacagtcga
caaaactcatctaacccagaatatgaaacgacctaagactatcagaattattttaagttt
tcaagttctttcttaaaatccaaaacttctttgattcaacaactttttactgaaacactc
atagcaaatcatctgcaaggacgaatccattgagaataatgtatactaatcatcatgtta
tagtaaaaattgcgtgatttgtacagtaaacggtcagcagcattgttgtgaggactaccc
ccttggattgttgcttgctctttgattgttgagcgggcatcgactggagctcggtatcga
ttgtgctatttacttaaacaactgatcgataaatatatttaaaatgtactcgctgccgcg
ttctccggaaaaaagggcaaatgtccgcatttcttcgtaaagtctggtgtttgcgtacac
ccatttttctttcattaaatatttataaatgcattgattcgcgtttgagtttcaaaatta
tctctatttgaaattccgcgcaaatctcatggacgcggcctaggattctcttcggtcgag
cgcttgcaacgacgctccgttctcccgcatgggtgttgtttagagtttgttcatattcca
aatatcggtcctatttttcagattttccagcgttttccaacatttttccacaattttcgg
agaaaattctgtaaaaattcaacttatctcgtttttccttcc

**Reverse Compliment**

ggaaggaaaaacgagataagttgaatttttacagaattttctccgaaaattgtggaaaaatgttggaaaacgctggaaaatctgaaaaataggaccgatatttggaatatgaacaaactctaaacaacacccatgcgggagaacggagcgtcgttgcaagcgctcgaccgaagagaatcctaggccgcgtccatgagatttgcgcggaatttcaaatagagataattttgaaactcaaacgcgaatcaatgcatttataaatatttaatgaaagaaaaatgggtgtacgcaaacaccagactttacgaagaaatgcggacatttgcccttttttccggagaacgcggcagcgagtacattttaaatatatttatcgatcagttgtttaagtaaatagcacaatcgataccgagctccagtcgatgcccgctcaacaatcaaagagcaagcaacaatccaagggggtagtcctcacaacaatgctgctgaccgtttactgtacaaatcacgcaatttttactataacatgatgattagtatacattattctcaatggattcgtccttgcagatgatttgctatgagtgtttcagtaaaaagttgttgaatcaaagaagttttggattttaagaaagaacttgaaaacttaaaataattctgatagtcttaggtcgtttcatattctgggttagatgagttttgtcgactgtgcaacagaaaggtttgggctttacattggaaaaagtgagtgtattgcatcggaagactatgtctggatacggagttgtgtttttgttagttttactgtaacatgatgattagtttacattattctctttggattcgtccttgcagagtatggactatgagtgtttcagtaaaaagttgttgaatcaaagaagttttggattttaagaaagaacttgaaaacttaaaatatctctgacagggttaggtcgtttcatattctgagttagatgagttttgtcgactgtgcaacagaaaggtttgggctttacattggaaaaagtgagagtattgccggtacaaaatctgccagcttcgacggaagggatagcttgtataaaacgaaaagttaaaggcaaaaccacaaatgatttttagcagttcttttttattcttctgcggatcgtcttgctttttcatgattgatgattcttggttctccatgattagtacggtaacacagcagcattttgtcaagctatgtttttgcgatga

| PROBE # | PROBE (5'-> 3') | PROBE POSITION * |
| --- | --- | --- |
| 2 | atattccaaatatcggtcctat | 89 |
| 7 | catttcttcgtaaagtctggtg | 294 |
| 8 | ctccggaaaaaagggcaaatgt | 319 |
| 9 | tatttaaaatgtactcgctgcc | 346 |
| 10 | gtatcgattgtgctatttactt | 388 |
| 11 | gtgatttgtacagtaaacggtc | 488 |
| 12 | caaggacgaatccattgagaat | 546 |
| 15 | cccagaatatgaaacgacctaa | 667 |
| 16 | ttgcacagtcgacaaaactcat | 693 |
| 17 | tttttccaatgtaaagcccaaa | 723 |
| 19 | caaaaacacaactccgtatcca | 774 |
| 21 | ttactgaaacactcatagtcca | 860 |
| 22 | tcaagttctttcttaaaatcca | 621 |
| 23 | taaccctgtcagagatatttta | 933 |
| 24 | ttgcacagtcgacaaaactcat | 693 |
| 26 | tttcgttttatacaagctatcc | 1073 |
| 28 | caagacgatccgcagaagaata | 1137 |
| 29 | cgtactaatcatggagaaccaa | 1180 |
| 30 | acatagcttgacaaaatgctgc | 1210 |

**2^nd^ exon sense**

| **PROBE #** | **PROBE (5'-> 3')** | **PROBE POSITION *** |
| --- | --- | --- |
| 1 | gtaacccaactagtctgaaa | 1 |
| 2 | aagtcaatttcgaccattct | 23 |
| 3 | atcttcttcgttctctttat | 45 |
| 4 | caacaaatcctggcggatcc | 70 |
| 7 | tggagaacgtcaggaacgag | 179 |
| 8 | ttcatcgatagttggtttct | 201 |
| 9 | gcgatgaagacgcaattgtc | 227 |
| 11 | tttggagttttccaagacgg | 271 |
| 12 | tccagaaccttcttcagtac | 293 |
| 13 | gaatgtagagcttcactgca | 325 |
| 14 | aagcaacctccttctggact | 350 |
| 17 | aactgagcggatcgttgatc | 440 |
| 18 | tccattcaaacttttcacgg | 462 |
| 19 | gtgtgattcttatcgaaagc | 485 |
| 20 | cttgaaagaacgggcggtga | 507 |
| 21 | gagcttcgagttgcttcata | 529 |
| 22 | tctggagttgtccagtgatc | 554 |
| 23 | aacgtcattataagcctgct | 576 |
| 25 | tgcgaactgatcacggcaac | 627 |
| 26 | ctccaagtttatcgtgggaa | 649 |
| 27 | gtgaacactccgacagttgg | 671 |
| 28 | tggatcatttcccttcatat | 693 |
| 29 | ttatcggcatctcctgcaag | 716 |
