# Supplementary material for: Heat shock in C. elegans induces downstream of gene transcription and accumulation of double-stranded RNA
Source: PLoS One. 2019 Apr 8;14(4):e0206715. doi: 10.1371/journal.pone.0206715 (PMC6453478; doi:10.1371/journal.pone.0206715)
Supplement: S3 File — (PDF) [file pone.0206715.s017.pdf]

Bioinformatics and Data analysis

Software used

List of software used... (html link if no citation available)
FastQC v0.11.7
Trimmomatic-0.36
STAR-2.5.2b
Microsoft Excel v15.30
Fiji/ImageJ v2.0.0 (https://imagej.net/Fiji)

1. In Python v3.6.6
   (a) biopython v1.71 (https://biopython.org/)
   (b) mygene v3.0.0 (https://pypi.org/project/mygene/)
   (c) goatools v0.8.4 (https://github.com/tanghaibao/goatools)
   (d) pandas v0.23.4 (https://pandas.pydata.org/)
   (e) seaborn v0.9.0 (https://seaborn.pydata.org/)
   (f) matplotlib v2.2.3 (https://matplotlib.org/)
   (g) matplotlib-venn v0.11.5 (https://matplotlib.org/)
2. In R v3.5.1
   (a) DESeq2 v1.20.0
   (b) Rsubread v1.28.1
   (c) ggplot2 v2.3.0 (https://ggplot2.tidyverse.org/)
   (d) dplyr v0.7.6 (https://www.rdocumentation.org/packages/dplyr/versions/0.5.0)
   (e) cowplot v0.9.3 (https://cran.r-project.org/web/packages/cowplot/vignettes/introduction.html)
   (f) magick v1.9 (https://cran.r-project.org/web/packages/magick/index.html)
   (g) here v0.1 (https://cran.r-project.org/web/packages/here/index.html)

Dogcatcher is available at...

https://github.com/Senorelegans/Dogcatcher

J2 enrichment version of Dogcatcher, scripts, and data to create all of the figures is available at...

https://github.com/Senorelegans/heatshock_and_tdp-1_dsRNA_scripts

FastQC and Trimming

Reads were verified for quality using FastQC, over-represented sequences and adapter contamination was trimmed using Trimmomatic-0.36 and then rechecked with FastQC. The list of adapters are in the attached file adaptertrim1ALL.fa Options were set to ILLUMINACLIP:adaptertrim1ALL.fa:2:30:10.

Aligning

Reads were aligned with star with the following commands
STAR –genomeDir /WS258/starindexesgenomic/STAR_WS258/
–readFilesIn ./replacedwordone.fastq
–runThreadN 12 –runMode alignReads
–limitBAMsortRAM 4133702715
–outReadsUnmapped Fastx
–outSAMattributes All
–outSJfilterCountUniqueMin 3 2 2 2
–outSJfilterCountTotalMin 5 3 3 3
–outSJfilterIntronMaxVsReadN 10 20 40
–alignIntronMin 10
–alignIntronMax 5000
–outFileNamePrefix ./starmapped/replacedwordone
–outSAMtype BAM SortedByCoordinate
–quantMode GeneCounts

Rsubread featureCounts

Genes and DoGs were assigned counts using the featureCounts package from RSubread with these options
featureCounts(files,
isGTFAnnotationFile = TRUE,
annot.ext = gtf,
GTF.attrType = gene_id
allowMultiOverlap = TRUE
strandSpecific = set to 1 or 2 for sense and antisense)

rRNA subtraction of read libraries for genes and DoGs

Input RNA was rRNA depleted before sequencing. However, due to low RNA yields, J2 immunoprecipitated RNA was not rRNA depleted before sequencing. To account for differences in Input-RNA rRNA depletion efficiency, we used the following subtraction procedure. Reads were mapped to a rRNA WS258.fasta genome created in biomart (http://uswest.ensembl.org/biomart/martview/). Genes and DoGs were normalized using the Ribosomal subtraction ratio derived from the equation below

$$L = I - (U + M)$$
$$\mu = \frac{\sum_{i=1}^{n} L}{n}$$
$$RSR = \frac{L}{\mu}$$
$$G = \frac{g}{RSR}$$

rRNA subtracted Library size for a sample (L) was calculated by taking input reads (I) minus the sum of the number of uniquely mapped reads to the rRNA fasta (U) and the number of multi-mapped reads to the rRNA fasta (M). The average library size (μ) is the sum of all the rRNA subtracted libraries divided by the amount of samples (n). The per sample ribosomal subtraction ratio (RSR) is calculated by dividing L by (μ). The rRNA normalized gene or DoG (G) is found by dividing the gene or DoG (g) by the RSR. Finally, we round g up to get an integer input for DESeq2. It is important to note that this equation was applied separately for Heatshock vs WT and tdp-1(ok803) vs WT, as well as within these groups separating input and J2 samples.

Ratio of ratios differential expression for genes and DoGs

We used the likelihood ratio test (LRT) option in DESeq2 for all analysis of J2 enrichment from input RNA. The LRT is used to find the difference between a full and reduced model.
full model = assay + condition + assay:condition
reduced = assay + condition
using the heat shock analysis as an example, the interaction term 'assay:condition' represents the ratio of the ratios:
(J2 for HS / Input for HS) / (J2 for NOHS / Input for NOHS)
or equivalently
(J2 for HS / J2 for NOHS) / (Input for HS / Input for NOHS)
The model will then show which genes are J2 enriched in heat shock compared to no heat shock. We did the same for tdp-1(ok803).
In this study, all enrichment differential expression was done with the following commands where the assay is enrichment and condition is heat shock, *tdp-1(ok803)* , or wild type worms.
dds = DESeqDataSetFromMatrix(countData = cts, colData = coldata, design= assay + condition + assay:condition)
dds$condition = factor(dds$condition, levels=c("C" ,"T"))
dds = DESeq(dds, test="LRT", reduced= assay + condition, parallel = TRUE)
For more information visit this post by DESeq2 creator Michael Love
https://support.bioconductor.org/p/61509/

Analysis of Brunquell 2016

Four samples were used in the analysis. Two heat shock (SRX1932621, SRX1932622) and two with no heat shock (SRX1932620, SRX1932619). All samples were processed similarly to our samples except for the DESeq2 step. Since there was no enrichment procedure we used the standard Dogcatcher algorithm and additional differential expression pipeline using the DESeq2 commands below.
dds = DESeqDataSetFromMatrix(countData = cts, colData = coldata, design= condition)
dds$condition = factor(dds$condition, levels=c("Heat shock" ,"Control"))
dds = DESeq(dds)

Dogcatcher

Dogcatcher uses a sliding window approach to calculate coverage. Bedgraph sense or antisense reads are assigned to these coverage windows and if coverage is above a set percentage threshold they will continue (meta read-through) or stop at the next gene on the same strand (local read-through). Downstream of gene transcripts (DoGs) use sense reads to the gene and slide 5' to 3' starting from the 3' annotated end. Antisense downstream of gene transcripts (ADoGs) use antisense reads to the gene and slide 5' to 3' starting from the 3' annotated end. Previous of gene transcripts (PoGs) use sense reads to the gene and slide 3' to 5' starting from the 5' annotated end. Antisense previous of gene transcripts (APoGs) use antisense reads to the gene and slide 3' to 5' starting from the 5' annotated end. PoGs are removed if they overlap a DoG. APoGs are removed if they overlap any ADoGs. ADoGs and APoGs are removed if they overlap DoGs, PoGs, or genes on the opposite strand. For improved normalization in DESeq2, non-significant genes are added when calculating differential expression. These non-significant genes are then removed directly after.
The J2 enriched version of Dogcatcher performs the Ribosomal Subtraction Ratio normalization on counts for RSubread as well as the LRT method from DESeq2 on normalized counts.

Antisense ratio for treatment over wildtype in input RNA(Fig3)

Counts were generated using RSubread. All genes were filtered to have a base mean over 20 in sense and antisense counts and log transformed. An antisense over sense ratio was then made for each condition. After this, a ratio was made of treatment over wild type.

Hyper-geometric distribution of gene overlaps for GO terms

The HYPGEOM.DIST equation was used in excel with the following parameters and the cumulative distribution set to false. Base mean, FDR, and log2 fold change is from the output of DESeq2 from sense and antisense for treatment over wild type.

population = all genes
overlap = significant genes (FDR <0.05) only in both comparisons
treatment 1 = significant genes only in heat shock
treatment 2 = significant genes only in *tdp-1(ok803)*

The hyper-geometric equations are in the supplemental HS_OK_HYPERGEODIST_and_overlap_LIST.xlsx

Removing DoGs or ADoGs overlapping with Operons

Operons were obtained from wormbase WS258.gff3 by writing out lines that contained “operon” and not “deprecated_operon”. 1388 operons were then matched to the longest DOGs and DOGs were removed if they had any overlap with operons on the same strand. Overlap was found by comparing the start or end coordinates of operons to the start or end of the longest DOG and removing if they were on the same strand. This filtering removed 67 and 36 Runon or operon overlaps from Heatshock and tdp-1(ok803) vs Wildtype comparisons. Similarly, ADOGs were removed if they had an operon overlapping on the opposite strand, none of the ADOGs in this study had operons on the opposite strand.

Terminal Inverted Repeats in DoGs

Terminal inverted repeats (TIR) were obtained from the WS258 tandem and inverted repeats gff. We first counted the amount of TIRs with any overlap to a DoG on the positive strand and divided by the length of the DoG. Next, we generated a background of downstream intergenic sequences for every gene on the positive strand. For the size of our random downstream intervals, we sampled without replacement from the length distribution of our significantly J2 enriched or depleted heat shock DoGs. If a random interval was larger than the distance to the next downstream gene the random interval was stopped at the next gene. We next sampled 50 of our random intervals and computed the mean 10,000 times. Finally, we used a student’s T-test and found significant differences (Padj < 0.016) comparing J2 enriched, J2 depleted, and non-significant percent coverage to random downstream intervals. We also found the T-test significant when comparing non-significant DoGs to J2 enriched and J2 depleted DoGs.

GO term analysis with mygene and goatools

The python package mygene was used to get entrez id’s for each gene or DoG for input into goatools. For obtaining genes inside of significant (fdr <0.05) GO terms related to translation we first obtained every significant GO term that contained the word ”translation”, ”ribosomal”, and ”ribosome”. We then took all of the significant genes and removed duplicates that belonged to multiple GO terms. Since goatools does not provide a background gene list for worms, one was created by downloading all genes with the taxid 6239 and creating a dictionary similar to the ones made in the goatools test data folder. You can download the list from NCBI with the link in genes_NCBI_6239_marko_ALL.py and it will create the dictionary when imported into another python script. Genes with overlap from DoGs on the opposite strand were obtained from the ”combined_DOG_with_biotypes_UNPACKED.csv” output from Dogcatcher. These were then combined with ADoGs to find all genes that could have significant antisense transcription starting from outside of the gene region. All genes, DoGs, PoGs, ADoGs, and APoGs, were filtered for a significance of padj < 0.05 and were considered ”up” in treatment vs control if they had a LFC > 0 and ”down” in treatment vs control if they had a LFC < 0.
